# SPR biosensor with a graphene overlayer for carcinoma detection

**DOI:** 10.3389/fbioe.2026.1789453

**Published:** 2026-02-23

**Authors:** Talia Tene, Katherine Tixi Gallegos, María José Mendoza Salazar, Lala Gahramanli, Rana Khankishiyeva, Cristian Vacacela Gomez

**Affiliations:** 1 Department of Chemistry, Universidad Técnica Particular de Loja, Loja, Ecuador; 2 Carrera de Ingeniería Química, Facultad de Ciencias, Escuela Superior Politécnica de Chimborazo (ESPOCH), Riobamba, Ecuador; 3 Facultad de Ciencias, Grupo de Investigación CIDED, Escuela Superior Politécnica de Chimborazo (ESPOCH), Riobamba, Ecuador; 4 Nano Research Laboratory, Center of Excellent, Baku State University, Baku, Azerbaijan; 5 Faculty of Physics, Chemical Physics of Nanomaterials, Baku State University, Baku, Azerbaijan; 6 Institute of Radiation Problems, Ministry of Science and Education of the Republic of Azerbaijan, Baku, Azerbaijan; 7 Department of Physics and Chemistry, Azerbaijan University of Architecture and Construction, Baku, Azerbaijan; 8 Department of Physics, University of Calabria, Rende, Italy; 9 Universidad Ecotec, Samborondón, Ecuador

**Keywords:** biosensor, carcinoma, copper, graphene, sensitivity, SPR, titanium dioxide

## Abstract

Early carcinoma detection benefits from label-free, high-sensitivity surface plasmon resonance (SPR) biosensors. We computationally evaluated multilayer SPR architectures based on CaF_2_/Cu/TiO_2_/Graphene using the transfer-matrix method at 633 nm. Across 1–5 ng/mL, we analyzed reflectance, resonance-angle shifts, and near-field profiles, and derived sensitivity, detection accuracy (DA), figure of merit, and the limit of detection (LOD). The CaF_2_/Cu/TiO_2_/Graphene stack yielded the best performance, achieving 481.29°/RIU sensitivity and DA = 0.80, with pronounced evanescent-field confinement at the sensing interface. Under identical modeling conditions, this graphene-integrated configuration outperformed TiO_2_-only and Cu-only baselines within the studied range. These results indicate a cost-effective platform for sensitive carcinoma biomarker detection. Calculation details for LOD and other metrics are provided in Methods, and practical considerations for experimental realization are discussed.

## 1 Introduction

Carcinomas constitute a major share of the global cancer burden, reinforcing the need for early detection at clinically relevant biomarker levels (World Health Organizat ion, 2023; Bray et al., 2024; Singal et al., 2023). In practice, the challenge is to identify tumor-associated targets at very low concentrations (often ng·mL^−1^) in complex biofluids while minimizing sample preparation and time to result (Trapé et al., 2011). These requirements motivate biosensor platforms that combine high sensitivity with stable, narrow resonances and robust operation under realistic assay conditions.

Surface plasmon resonance (SPR) enables real-time, label-free monitoring of biomolecular binding at functionalized metal–dielectric interfaces in the Kretschmann prism configuration (Wang et al., 2025; Pandey et al., 2022; Shukla et al., 2022; Chirico et al., 2025). Under TM-polarized illumination, small refractive-index perturbations within the evanescent field translate into measurable shifts of the resonance angle or wavelength (Pandey et al., 2022; Shukla et al., 2022), which supports early cancer detection and quantitative affinity analysis (Wang et al., 2025; Chirico et al., 2025).

Translating SPR to ultralow-concentration carcinoma detection demands continued gains in sensitivity, detection accuracy, and robustness under realistic assay conditions (Bellassai et al., 2019). Materials engineering at the detection interface—modifying plasmonic metals and dielectric/2D overlayers—can enhance near-field intensity, increase analyte capture, reduce optical attenuation, and improve chemical stability (Sasidevi et al., 2025). Critical reviews emphasize that the choice and order of plasmonic metals, dielectric spacers, and 2D materials strongly influence coupling efficiency, field confinement, and figure of merit (Cho et al., 2025). Recent work spans biomarker and cancer-cell detection (including photonic-crystal-fiber implementations) and explores AI-assisted optimization for SPR design (Shakya et al., 2026; Ramola et al., 2026; Ramola et al., 2025a; Ramola et al., 2025b; Ramola et al., 2025c; Ramola et al., 2025d; Shakya and Singh, 2023).

Copper (Cu) is attractive for cost-sensitive plasmonic biosensing due to its abundance and strong visible-range resonances (Xin et al., 2021; Indhu et al., 2024), but it readily oxidizes in ambient and aqueous environments, degrading resonance quality (Hosseinpour and Johnson, 2017). A monolayer-scale graphene overlayer acts as an ultrathin diffusion barrier that stabilizes Cu while preserving optical thickness (García de Abajo, 2014); experiments show graphene-protected Cu films retain excellent plasmonic performance over extended periods, even under humid or corrosive conditions (García de Abajo, 2014; Wu et al., 2020), supporting repeatable calibration and reliable detection of small refractive-index perturbations at low analyte levels in carcinoma assays (Kravets et al., 2014).

Titanium dioxide (TiO_2_) complements this design by providing chemical stability, biocompatibility, and a comparatively high refractive index (Yesudasu et al., 2023). Inserted as a nanometric dielectric spacer, TiO_2_ improves impedance matching and strengthens near-field confinement, increasing the resonance shift for a given refractive-index change (Basit et al., 2025). These properties benefit carcinoma biomarker detection, where higher field intensity at the biointerface enhances transduction at low surface coverage and lowers the effective detection limit (Zhang et al., 2018).

Beyond copper protection, graphene offers a functional, high-surface-area interface that supports dense immobilization of biorecognition elements and facilitates π–π interactions with aromatic and π-conjugated biomolecules, improving capture within the plasmonic “hot zone” (Butt, 2025). Mechanistically, the two-dimensional conductive sheet modifies the electromagnetic boundary conditions at the metal–dielectric interface, slightly hybridizes the metal surface plasmon, and pulls the evanescent-field maximum toward the analyte side—effects reported to strengthen near-field interaction and sensitivity in graphene-assisted SPR architectures, including cancer-related detection contexts (Butt, 2025; Mostufa et al., 2022).

Within this materials framework, the CaF_2_/Cu/TiO_2_/graphene stack is synergistic: CaF_2_, a low-index prism material, provides efficient momentum matching and convenient angular interrogation; Cu supplies strong plasmonic excitation and cost advantages; TiO_2_ improves confinement as a thin spacer; and graphene furnishes a stable, functional biointerface while protecting Cu. We explicitly note the trade-off inherent to adding lossy overlayers: excessive graphene thickness or non-optimal optical parameters can broaden the resonance (FWHM increase) and reduce detection accuracy (DA). Accordingly, we adopt a single-layer graphene overlayer as a practical compromise that maintains a narrow resonance while increasing the fraction of modal energy residing in the analyte. The detection stack is prism-coupled using CaF_2_ for efficient momentum matching and robust monitoring of resonance-angle shifts (Xu et al., 2024).

Building on multilayer SPR designs that balance field concentration and linewidth (Cho et al., 2025; Mostufa et al., 2022)—and the broader trends summarized above (Shakya et al., 2026; Ramola et al., 2025a; Ramola et al., 2025c)—we present a comparative, simulation-based optimization of CaF_2_/Cu/TiO_2_/graphene architectures via transfer-matrix modeling at 633 nm. We systematically tune layer thicknesses to concentrate the evanescent field at the sensing interface while preserving resonance sharpness and stability, and we report quantitative performance metrics and tolerance analyses relevant to fabrication variability. The study provides design guidance for cost-effective, high-sensitivity SPR platforms intended for carcinoma biomarkers at ng·mL^−1^ levels.

## 2 Materials and methods

This section details the structural configuration of the proposed SPR biosensor architectures, along with the computational modeling and performance evaluation strategies applied throughout this study. The analysis focuses on multilayer designs incorporating CaF_2_, Cu, TiO_2_, and G, with the aim of evaluating their combined impact on plasmonic resonance behavior under ultralow concentration conditions, relevant for the detection of carcinoma biomarkers. Using TMM (see Supplementary Material), we simulated reflectance spectra and evaluated critical sensor parameters, such as the resonance angle shift at a working wavelength of λ = 633 nm.

### 2.1 Performance metrics and optimization protocol

The sensor’s performance was evaluated using a set of key optical descriptors, including angular sensitivity (Sθ), full width at half maximum (FWHM) of the SPR tilt, figure of merit (FoM), and limit of detection (LoD). All simulations were performed under transverse magnetic polarization (TM). Angular sensitivity Sθ represents the change in the resonance angle (θ_spr_) in response to variations in the analyte’s refractive index (n_a_) and was calculated using finite differences around a reference index (n_0_) with a small perturbation step, ensuring numerical stability and local linearity of the response.

The FWHM was determined by fitting the reflectance curve R(θ) near the resonance minimum, allowing for precise determination of the resonance sharpness. To quantify the balance between sensitivity and resonance sharpness, the FoM was defined as FoM = Sθ/FWHM (units: RIU^−1^). The limit of detection (LoD) was estimated based on Sθ and the measurement resolution, providing a practical indicator of the sensor’s ability to detect subtle changes in analyte concentration.

For greater comprehensiveness, two complementary descriptors were calculated when they offered additional information: detection accuracy (DA), defined as DA = 1/FWHM (degrees^-1^), and quality factor (QF), expressed as QF = θ_spr_/FWHM (dimensionless). However, Sθ, FWHM, FoM, and LoD were emphasized as the primary comparative metrics, while DA and QF are reported in the Supplementary Material where applicable.

To ensure fair comparisons between multilayer configurations, the incidence angle θ was scanned across the range encompassing the critical angle and the SPR region using a coarse-to-fine angular resolution strategy, followed by high-resolution interpolation to determine θ_spr_ with submillimeter accuracy. Layer thicknesses, particularly for the metallic (Cu) and dielectric (TiO_2_) films, were optimized using a discrete grating search to maximize the FoM at the reference index n_0_.

Low-concentration carcinoma detection conditions were modeled by simulating small increments in the refractive index around n_0_. The corresponding metrics (S_θ_, FWHM, FoM, and LoD) for the various configurations are summarized in Supplementary Tables S17–S19. To reflect realistic serum/plasma workflows, performance was benchmarked around 1–5 ng·mL^−1^, consistent with commonly used cut-offs for CEA, CYFRA 21-1, and SCC-Ag and with standard dilution ratios in label-free assays (Hall et al., 2019; Okamura et al., 2013; Oh and Bae, 2018; Calvo-Lozano et al., 2022).

### 2.2 Transfer-matrix modeling (TMM)

The reflectance spectrum R(θ) was calculated using the transfer matrix method (TMM), a well-established formalism for the optical analysis of layered media (Wu et al., 2010; Tene et al., 2025a). This approach involves the sequential construction of individual layer matrices based on the boundary conditions of the tangential electromagnetic field, followed by multiplication to obtain the overall transfer matrix and the corresponding Fresnel reflection coefficient.

The complete derivation, including expressions for electric and magnetic field continuity, layer-specific propagation matrices, the formulation of the total system matrix, and the final reflectance calculation, provided in the Supplementary Numerical Modeling section (Supplementary Section S1; Supplementary Equations S1–S7). A representative SPR resonance curve and a validation of the model against existing multilayer references in the literature are presented in Supplementary Figure S1 (Cheon et al., 2014). All simulations were performed under transverse magnetic polarization (TM), consistent with the excitation conditions for surface plasmon resonance in Kretschmann-type configurations (Rumi et al., 2024; Rafi et al., 2025). Layer thicknesses were identified by comparing the discrete configurations reported in Sections 3.2–3.4. The selection criterion prioritized a high FoM = S_RI_/FWHM together with a high DA = Δθ/FWHM and moderate attenuation. The reported “optimized” values correspond to candidates that outperformed their neighboring tested points in these metrics across the 1–5 ng·mL^−1^ window.

### 2.3 Configurations under study

Supplementary Scheme S1 provides an overview of the architectures analyzed, fixing the stacking order and the parameters compared throughout the study. The incident beam enters through the prism and is swept over the angle θ until the surface plasmon mode is excited, as indicated in Supplementary Scheme S1. A continuous metal film is deposited on the exit face of the prism, and its thickness is optimized in each case because it simultaneously governs the coupling efficiency and the resonance linewidth; this relationship produces the reflectance minimum visible along the path marked. Between the metal and the analyte, an ultrathin TiO_2_ layer is inserted to shape the evanescent-field profile without incurring excessive losses, a function represented as a thin dielectric band immediately above the metal.

The top sensing layer includes graphene (G), graphene oxide (GO), reduced graphene oxide (rGO), or single-walled semiconducting carbon nanotubes (sSWCNTs), used individually as ultrathin layers. These 2D materials enhance near-field interaction at the sensor-analyte interface due to their high surface area. The sensing region is modeled as a biofluid containing carcinoma-associated biomarkers at concentrations ranging from 1 to 5 ng/mL, corresponding to small changes in the local refractive index. All optical constants (n, k) and nominal thicknesses of the prism materials, the Cu and TiO_2_ layers, the 2D nanomaterial coatings, and the analyte medium at 633 nm are detailed in Supplementary Table S2, along with bibliographic sources (Akib et al., 2024; Sayed et al., 2025; Xue et al., 2013; Tene et al., 2025b; Song et al., 2020; Hossea and Rugumira, 2024; Rafighirami et al., 2025).

This work is a computational modeling study based on the transfer-matrix method; no experimental fabrication or surface/structural characterization was performed. To support reproducibility and guide future prototypes, Section 4 outlines an XPS/XRD verification protocol to confirm layer chemistry (graphene C1s, Cu oxidation state, Ti 2p) and crystalline phase/texture prior to optical benchmarking.

### 2.4 Performance metrics and detection limit

We quantify performance using the definitions already given in Equations 1–5: the refractive-index sensitivity S_RI_ = Δθ/Δn (1), detection accuracy DA = Δθ/FWHM (2), quality factor QF = S_RI_/FWHM (3), figure of merit FoM = S_RI_(1−R_min_) (4), and the combined sensitivity factor (5) (Wu et al., 2010; Tene et al., 2025a).

Refractive-index sensitivity (angular readout):

$$S_{RI}=\frac{\Delta \theta }{\Delta n}$$ (1)

Detection accuracy (DA):

$$DA=\frac{\Delta \theta }{FWHM}$$ (2)

(Δθ in degrees; FWHM in degrees; DA is dimensionless.)

Quality factor (QF):

$$QF=\frac{S_{RI}}{FWHM}$$ (3)

Figure of Merit (FoM):

$$FoM=\frac{S_{RI}\left(1-R_{\min}\right)}{FWHM}$$ (4)

with R_min_ the minimum normalized reflectance at resonance.

Combined Sensitivity Factor (CSF):

$$CSF=\frac{S_{RI}\times \left(R_{\max}-R_{\min}\right)}{FWHM}$$ (5)

where R_max_ is the normalized reflectance before resonance (non-resonant angle/wavelength).

We define the minimum resolvable angular change δθ_min_ as the effective angular step of the algorithm. From Equation 1, the smallest resolvable refractive-index change follows by setting Δθ = δθ_min_; therefore:

$${LoD}_{n}=\frac{{\delta \theta }_{\min}}{S_{RI}}$$ (6)

In our baseline simulations (Equation 6), we adopt δθ_min_ = 0.005°, hence LOD_n_ = 0.005°/S_RI_. Full TMM definitions and notation are provided in the Supplementary Material.

## 3 Results and discussions

### 3.1 Prism material selection and optimization

**FIGURE 1:**
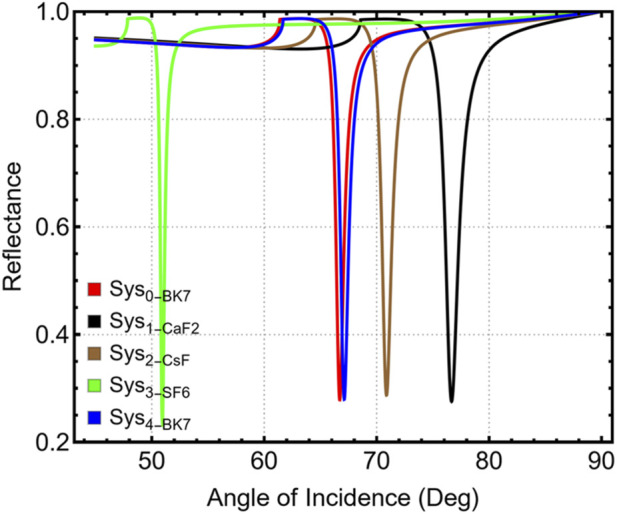
Simulated angular reflectance curves for systems with different prism materials (Sys_0_–Sys_4_) under TM-polarized light at 633 nm.

The performance of the SPR biosensor array was evaluated using five solid-state prism materials (BK_7_, CsF, BaF_2_, CaF_2_, and Schott N-SF6 glass). The objective was to identify the prism substrate that provides the most favorable conditions for detecting low-concentration carcinoma biomarkers using angular interrogation. All simulations were performed under TM polarization at λ = 633 nm. CaF_2_ proved to be the most suitable prism material, offering the best balance between resonance-angle positioning, sensitivity enhancement, angular displacement, and resonance sharpness, while also ensuring experimental feasibility. Figure 1 shows the simulated angular reflectance curves for the five prisms; the RI–θ_SPR_ trend is provided in Supplementary Figure S2.

**FIGURE 2:**
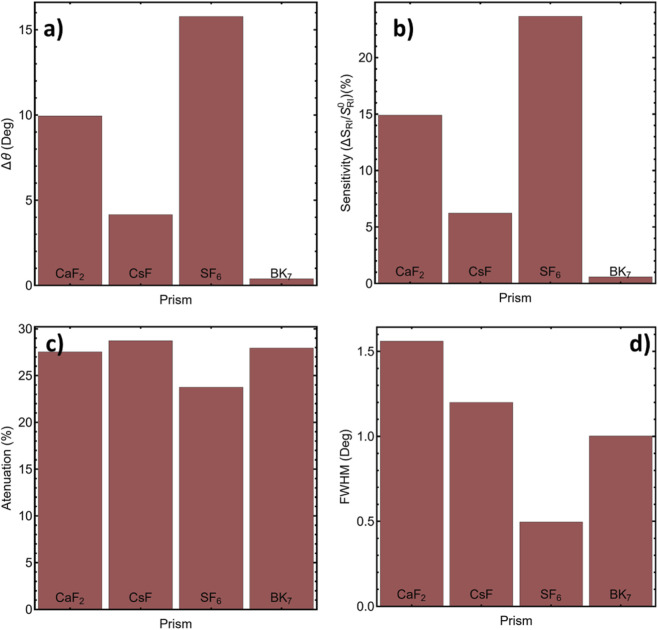
Comparison of prism materials in terms of: **(a)** Angular shift (Δθ); **(b)** Sensitivity enhancement (%); **(c)** Reflectance attenuation (%); and **(d)** FWHM (°).

For performance comparison, we extracted four metrics: angular displacement (Δθ), sensitivity improvement, reflectance attenuation, and full width at half maximum (FWHM). As summarized in Supplementary Table S3 and Figure 2, CaF_2_ achieves a substantial angular displacement (Δθ = 9.94°) and sensitivity improvement (14.90%), ranking second to N-SF6 (Δθ = 15.78°; FWHM = 0.49°). Despite the strong optical response of SF_6_, CaF_2_ offers a more practical balance for experimental SPR platforms (commercial availability in high optical quality, broad transparency, and manageable θSPR window), while maintaining acceptable attenuation (27.53%) and a narrow line (FWHM = 1.56°). In contrast, BK_7_ shows a small angular shift (Δθ = 0.39°) and minimal sensitivity gain, making it suboptimal for low-concentration detection. Based on this overall balance, CaF_2_ was selected as the prism for subsequent simulations and sensor evaluations.

For detection performance, we evaluated four metrics—angular displacement (Δθ), sensitivity enhancement, reflectance attenuation, and FWHM—summarized in Figures 2a–d and Supplementary Table S3. CaF_2_ shows a large angular shift (Δθ = 9.94°) with 14.90% sensitivity enhancement, 27.53% attenuation, and a narrow line (FWHM = 1.56°), providing the best practical balance. Schott N-SF6 glass attains the highest Δθ (15.78°) and the narrowest FWHM (0.49°), whereas BK_7_ exhibits minimal Δθ (0.39°) and sensitivity gain. We therefore adopt CaF_2_ for subsequent simulations and evaluations (see exact values in Supplementary Table S3).

### 3.2 Optimization of the plasmonic metal layer

**FIGURE 3:**
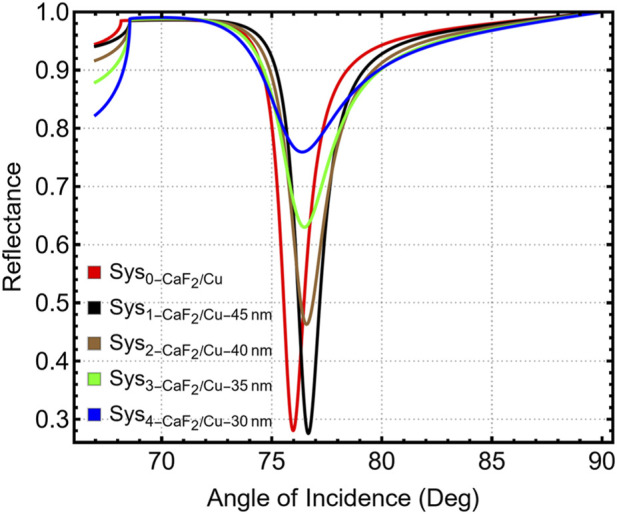
Reflectance spectra for CaF_2_-coupled SPR systems with varying Cu thicknesses (30–45 nm).

Following the selection of the prism material, the plasmonic metallic layer was optimized to maximize the sensitivity and angular performance of the SPR biosensor. Copper (Cu) was chosen due to its favorable visible-range response, low cost, and compatibility with graphene-based protective coatings. The optimal Cu thickness was identified by simulating five multilayer configurations using the CaF_2_ prism with Cu films of 30, 35, 40, and 45 nm, as well as a reference system without specified thickness (Sys_0_–Sys_4_). Figure 3 shows that increasing the Cu thickness progressively narrows and deepens the resonance. The corresponding change in resonance angle across thicknesses is provided in Supplementary Figure S3, where θ_SPR_ increases from 76.39° at 30 nm to 76.66° at 45 nm.

**FIGURE 4:**
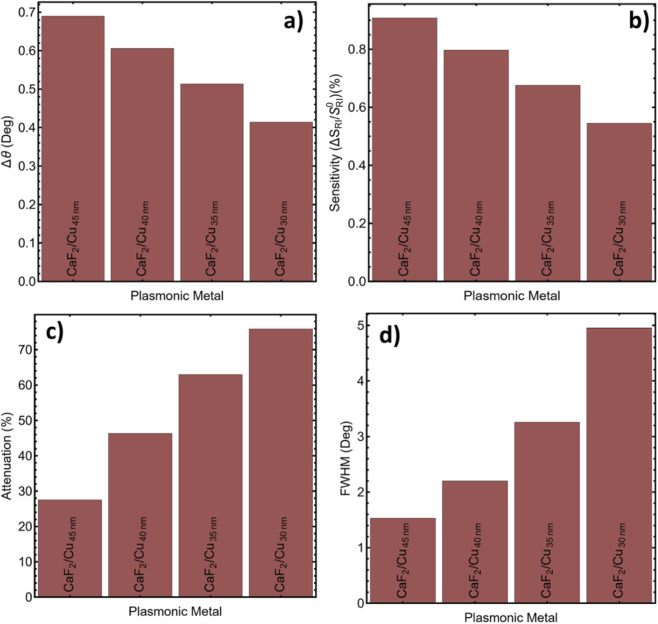
Performance metrics for CaF_2_/Cu_th_ SPR systems: **(a)** Angular shift (Δθ), **(b)** sensitivity enhancement (%), **(c)** reflectance attenuation (%), and **(d)** FWHM (°).

The quantitative comparison of performance metrics is presented in Supplementary Tables S5, S6. The CaF_2_/Cu_45_nm configuration achieves the greatest angular shift (Δθ = 0.69°) and the maximum sensitivity improvement (0.90%) when exposed to a simulated refractive index perturbation associated with carcinoma biomarkers (1 ng/mL). These values are supported by the trends shown in Figures 4a,b, where both Δθ and sensitivity increase with thickness. The improved detection performance at 45 nm can be attributed to greater surface plasmon confinement and a larger interaction volume near the detection interface. The 45 nm Cu layer provides sufficient field penetration and signal depth to transduce these subtle variations into measurable angular shifts.

Attenuation and resonance amplitude were also evaluated. As expected, reflectance attenuation increases with decreasing Cu thickness due to weaker coupling and higher radiative losses. The CaF_2_/Cu_45_nm system maintains a moderate attenuation of 27.53%, as shown in Figure 4c, indicating efficient plasmon excitation with acceptable signal strength. This configuration exhibits the narrowest full width at half the maximum (1.52°) among the tested systems (Figure 4d), enabling high angular resolution and a better signal-to-noise ratio. Conversely, thinner Cu layers resulted in reduced performance. The CaF_2_/Cu_30_nm system shows a minimum Δθ (0.41°), reduced sensitivity (0.54%), high attenuation (75.91%), and a full width-to-magnitude (FWHM) of 4.95°, limiting its usefulness for accurate low-concentration detection. Intermediate thicknesses (35–40 nm) offer better results than the thinnest layer but are still below the optimal 45 nm setting. Supplementary Table S7 provides further validation, confirming the consistency of SPR peak positions across the various configurations using the real part of Cu’s refractive index. All values remain tightly clustered around 76.5°, ensuring that the resonance remains within the detectable angular range while simultaneously optimizing sensitivity. The selected t_Cu_ outperforms neighboring tested thicknesses, yielding higher FoM with a narrower FWHM.

### 3.3 Optimization of the titanium dioxide layer for enhanced plasmonic sensing

TiO_2_ was integrated as a dielectric spacer between the plasmonic copper layer and the analyte medium to modulate the optical field distribution and improve sensor sensitivity. Its high refractive index, chemical stability, and compatibility with functional surfaces make it a valuable component in multilayer SPR platforms, especially when probing low-concentration biomolecular interactions. To evaluate the influence of TiO_2_ thickness on SPR performance, five multilayer configurations were simulated with fixed prism (CaF_2_) and copper layer (45 nm) parameters, varying the TiO_2_ spacer from 2 to 32 nm. The corresponding systems (Sys_0_–Sys_4_) were analyzed using angular reflectance spectra and key detection metrics.

**FIGURE 5:**
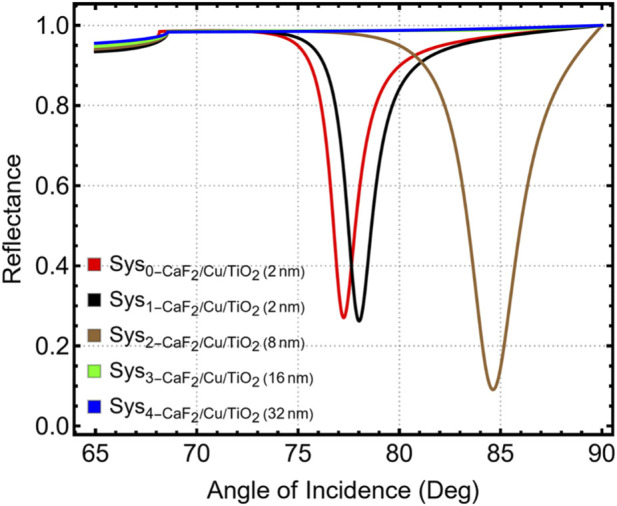
Reflectance spectra for CaF_2_/Cu systems with varying TiO_2_ thicknesses (2–32 nm).

Figure 5 shows that changes in TiO_2_ thickness significantly alter the reflectance profile. The sharpest and deepest resonance occurs for CaF_2_/Cu/TiO_2_-8 nm, indicating improved surface-plasmon coupling. The corresponding shift of the SPR angle as a function of spacer thickness is provided in Supplementary Figure S4: the resonance angle increases from 78.01° at 2 nm to 84.62° at 8 nm, and then decreases to 65.00° at 16 and 32 nm, reflecting the change in field-confinement behavior at larger spacer thicknesses.

**FIGURE 6:**
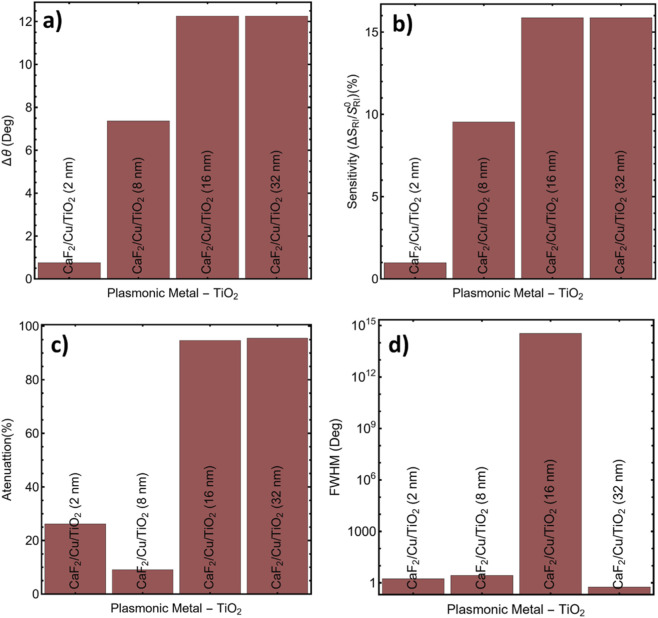
Performance metrics for SPR systems using different TiO_2_ spacer thicknesses: **(a)** Δθ, **(b)** Sensitivity, **(c)** Attenuation, and **(d)** FWHM.

The quantitative results summarized in Supplementary Table S9 and visualized in Figure 6 reinforce the optical observations. Δθ shown in Figure 6a reached 7.36° for 8 nm, significantly higher than the 0.75° for 2 nm. While larger shifts were obtained for 16 and 32 nm (both 12.25°), this came at the expense of resonance quality and signal stability. The sensitivity improvement (Figure 6b) followed a similar trend: the 8 nm system achieved an efficiency of 9.53%, outperforming the thinner coatings and offering a solid balance between response and optical quality. The attenuation behavior reflected in Figure 6c confirms this balance. While ultrathin TiO_2_ (2–8 nm) maintained low attenuation values, thicker films caused substantial losses, reaching 94.77% and 95.53% at 16 and 32 nm, respectively. The resulting over-attenuation suppresses reflectance modulation and produces resonance dips unusable for practical biodetection. The FWHM analysis, which can be seen in Figure 6d, showed a marked contrast in performance. The 8 nm configuration maintained a manageable FWHM of 2.71°, while the 16 nm system produced a drastically broad and unstable response, highlighting field delocalization and poor resonance sharpness.

Supplementary Table S10 corroborates the angular positioning behavior with consistent SPR peak positions for each TiO_2_ thickness. While the 16 and 32 nm layers exhibited the largest angular shifts, their resonance widths and high attenuation make them ineffective for stable biodetection in the ng·mL^-1^ range. In contrast, the 8 nm TiO_2_ layer demonstrated high sensitivity, acceptable sharpness, and strong resonance modulation, making it optimal for detecting subtle changes in refractive index associated with early-stage carcinoma biomarkers.

### 3.4 Optimization of nanomaterial overlayers for SPR biosensing

To improve the plasmonic performance of the CaF_2_/Cu/TiO_2_-based SPR biosensor, nanomaterial overlayers were integrated onto the titanium-dioxide surface. We evaluated ultrathin graphene (G), graphene oxide (GO), reduced graphene oxide (rGO), and single-walled semiconducting carbon nanotubes (sSWCNTs) and quantified their impact on SPR signal quality, sensitivity, and detection performance under carcinoma-biomarker conditions.

**FIGURE 7:**
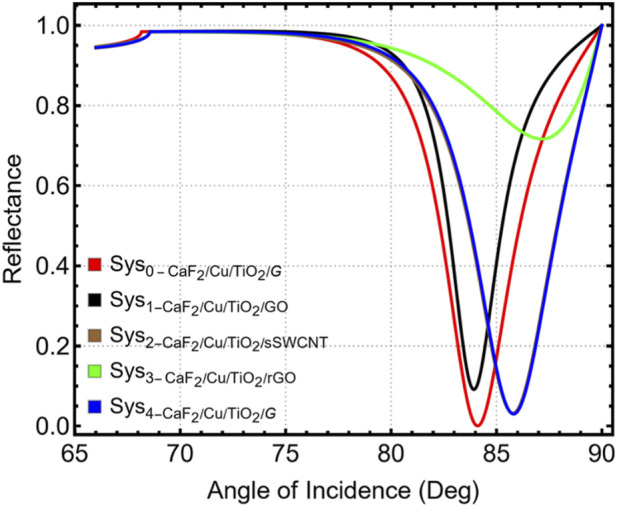
Simulated reflectance curves for nanomaterial-functionalized SPR configurations.

Figure 7 shows the reflectance spectra for all nanomaterial-functionalized systems (Sys_0_–Sys_4_), where the depth, sharpness, and angular position of the resonance vary with the optical properties of the top layer. Among the materials analyzed, the CaF_2_/Cu/TiO_2_/G configuration exhibits the most favorable spectral profile, with a sharp, well-defined resonance and minimal side-lobe distortion. The corresponding dependence of the SPR angle on the real part of the overlayer refractive index is provided in Supplementary Figure S5, illustrating how different top layers modify the plasmonic coupling condition.

**FIGURE 8:**
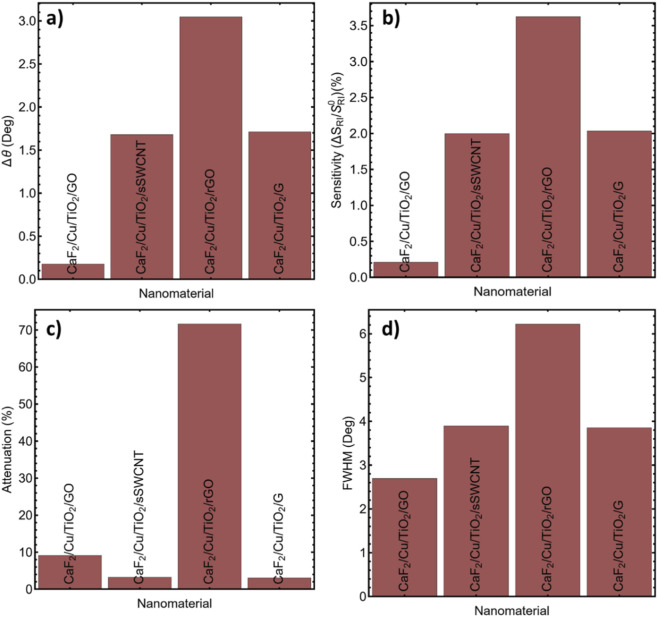
Performance comparison of nanomaterial overlayers integrated into the CaF_2_/Cu/TiO_2_ SPR architecture, were evaluated based on, **(a)** Δθ, **(b)** Sensitivity, **(c)** Attenuation, and **(d)** FWHM.

The results comparing the effects of the nanomaterials are summarized in Supplementary Tables S11–S13 and graphically represented in Figure 8; the refractive index properties used to model each nanomaterial are listed in Supplementary Table S13. G exhibited a strong angular shift (Δθ = 1.71°) and a 2.03% sensitivity improvement, surpassing GO and rGO, and showing a close resemblance to sSWCNTs. The Δθ and sensitivity improvement are graphically represented in Figures 8a,b, confirming that graphene and sSWCNTs offer the greatest refractive index responsiveness under identical biofluid conditions. Although rGO showed a slightly higher Δθ (3.02°) and sensitivity (3.62%), it also exhibited considerable disadvantages in signal attenuation and resonance amplitude. As observed in Figure 8c, the rGO-modified structure exhibits excessive attenuation (71.66%) and degraded spectral contrast, which may compromise detection reliability; its resonance curve broadened considerably, with a FWHM of 6.21°, as shown in Supplementary Table S12 and Figure 8d. G, on the other hand, maintained low attenuation (30.1%) and a narrower FWHM of 3.85°, providing a sharper and more stable resonance, suitable for biodetection. The GO layer showed the worst performance in terms of Δθ (0.17°), sensitivity (0.20%), and attenuation (9.14%), although it maintained a moderately sharp resonance (FWHM of 2.69°). The rGO layer, while optically active, exhibited excessive losses for practical detection, likely due to a lack of homogeneity and surface roughness, leading to plasmonic damping.

Taken together, these results underscore a sensitivity–linewidth balance introduced by lossy overlayers. Although adding a 2D overlayer can raise S_RI_ and Δθ, excessive absorption broadens the resonance (FWHM increase) and erodes detection accuracy DA = Δθ/FWHM as well as FoM = S_RI_/FWHM. Consequently, we adopt a single-layer graphene implementation as a practical configuration that increases S_RI_ while preserving a narrow resonance and acceptable attenuation.

The results obtained demonstrate that G is the optimal nanomaterial layer for this SPR architecture. The CaF_2_/Cu/TiO_2_/G configuration successfully combines angular sensitivity, spectral sharpness, and low signal loss, creating favorable conditions for high-resolution detection of carcinoma biomarkers at low concentrations. Graphene’s inherent chemical stability and biocompatibility make it ideal for repeatable detection in complex biological environments. This improvement to the nanomaterial provides a critical performance gain for the sensor system, directly contributing to lowering the detection limit and improving reproducibility, key aspects in early-stage cancer diagnosis. In line with this balance, the monolayer choice maximizes FoM while maintaining high DA, ensuring robust angular readout under carcinoma assay conditions. A single graphene sheet strengthens the near-surface field and enhances index-to-angle transduction while keeping the resonance linewidth tight; thicker or lossier overlayers were avoided to preserve angular acuity.

### 3.5 Sensor performance

The efficacy of an optimized SPR biosensor for biomedical detection, particularly for the detection of carcinoma biomarkers, was evaluated. The complete CaF_2_/Cu/TiO_2_/G system was analyzed under different carcinoma concentrations, from 1 to 5 ng/mL. The system incorporates all previously optimized components: a CaF_2_ prism for increased coupling efficiency, a 45 nm copper layer for strong plasmonic excitation, an 8 nm titanium dioxide intermediate layer for field enhancement, and a G top layer to facilitate biomolecule adsorption and promote signal transduction.

**FIGURE 9:**
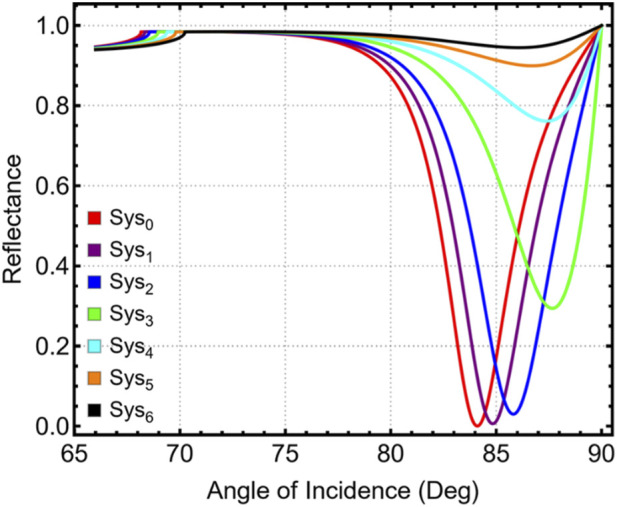
SPR reflectance curves of the CaF_2_/Cu/TiO_2_/G biosensor at varying carcinoma concentrations (1–5 ng/mL), demonstrating distinct resonance angle shifts indicative of enhanced biosensing performance.

Figure 9 shows the reflectance profiles of the CaF_2_/Cu/TiO_2_/G structure at different carcinoma concentrations, along with the deionized water (DW) reference system. A clear angular shift in the SPR curve is observed with increasing analyte concentration, confirming the biosensor’s sensitivity to refractive index variations induced by the presence of carcinoma. The sharpest resonance minimum and the most pronounced shift were recorded at 5 ng/mL, indicating the system’s ability to detect even small changes in biological analyte levels.

**FIGURE 10:**
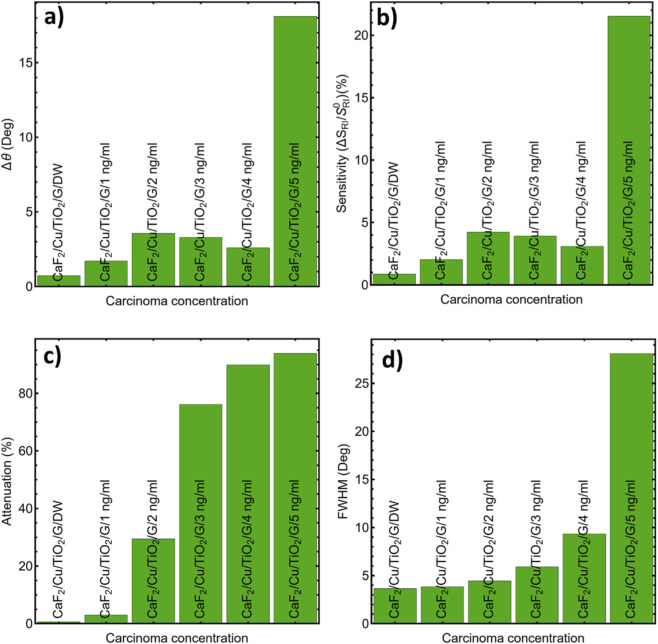
Evaluation of the sensor’s performance metrics as a function of carcinoma concentration: **(a)** Δθ, **(b)** Sensitivity, **(c)** Attenuation, and **(d)** FWHM.

The performance results are summarized in Supplementary Table S16. The sensor shows a progressive increase in angular displacement (Δθ) from 0.72° at 0 ng/mL (DW) to 18.10° at 5 ng/mL carcinoma, as shown in Figure 10a. This significant displacement indicates excellent angular resolution and high detectability. Similarly, sensitivity, expressed as the percentage change in reflectance per unit change in IR, increases from 0.86% to 21.52% across the entire concentration range (Figure 10b). The attenuation values shown in Figure 10c also increase with concentration, reaching up to 93.97% at 5 ng/mL. This marked attenuation of the reflectance signal improves the signal-to-noise ratio, although extremely high attenuation levels can hinder accurate detection due to the reduced reflected intensity. It is important to note that a balance must be struck between attenuation and measurement clarity. For example, at concentrations of 3 ng/mL or higher, the reduction in signal intensity becomes significant, requiring high-resolution instrumentation for accurate detection.

Full-width half-maximum (FWHM) values (Figure 10d) range from 3.65° (in dilute water) to 28.11° at 5 ng/mL, indicating a steeper SPR slope with increasing analyte concentration. While wider resonances may suggest a larger detection volume, they also reduce detection sharpness and can decrease accuracy in systems with overlapping signals or background noise. Therefore, although the sensor achieves a high FWHM at the highest concentration, the optimal working range for both sensitivity and resolution is around 2–3 ng/mL, where the FWHM remains below 6° and attenuation is manageable. Supplementary Table S17 further correlates the RI values of carcinoma concentrations with the SPR peak positions. The peak position shifts from 84.83° at 1.3317 (DW) to 66.00° at 1.3485 (5 ng/mL), providing further evidence of the index’s high sensitivity. The correlation is consistent and monotonic, supporting the reliability of the angle interrogation method for quantitative biodetection.

The advantages of this system include its high angle shift, excellent sensitivity, and reliable response to small variations in the refractive index, making it suitable for the early detection of carcinomas. Its layered design, which utilizes copper, TiO_2_, and graphene, synergistically contributes to field confinement, signal enhancement, and biochemical interaction. On the other hand, the main limitations relate to high attenuation and resonance broadening at higher concentrations, which may necessitate more precise detection instruments. Furthermore, the nonlinear growth of FWHM could pose interpretive challenges in real-time monitoring applications unless compensated for with advanced signal processing techniques. This balance motivates the use of monolayer graphene: it maintains a sharp resonance while providing the sensitivity gain required near clinically relevant concentrations.

### 3.6 Sensor metrics

The practical efficiency of the proposed CaF_2_/Cu/TiO_2_/G SPR biosensor was evaluated for carcinoma detection using quantitative performance metrics: sensitivity (S), detection accuracy (DA), quality factor (QF), figure of merit (FoM), limit of detection (LoD), and signal contrast factor (CSF). These metrics allow for a comprehensive evaluation of the sensor’s response to increasing concentrations of carcinoma biomarkers, from 1 to 5 ng/mL, modeled as changes in refractive index (RI), as shown in Supplementary Table S18. We interpret all metrics within a sensitivity–linewidth balance: increases in S_RI_ are only beneficial if FWHM remains sufficiently narrow. By definition, DA = Δθ/FWHM and FoM = S_RI_/FWHM; both penalize resonance broadening. The 1–5 ng·mL^-1^ window brackets common decision thresholds used in serum for CEA (∼5 ng·mL^-1^), CYFRA 21-1 (∼3–4 ng·mL^-1^), and SCC-Ag (∼2 ng·mL^-1^), aligning our modeled index steps with clinically actionable ranges; typical assay dilutions (≈1:10–1:50) keep the sensor-side concentrations within this window (Hall et al., 2019; Okamura et al., 2013; Oh and Bae, 2018; Calvo-Lozano et al., 2022).

**FIGURE 11:**
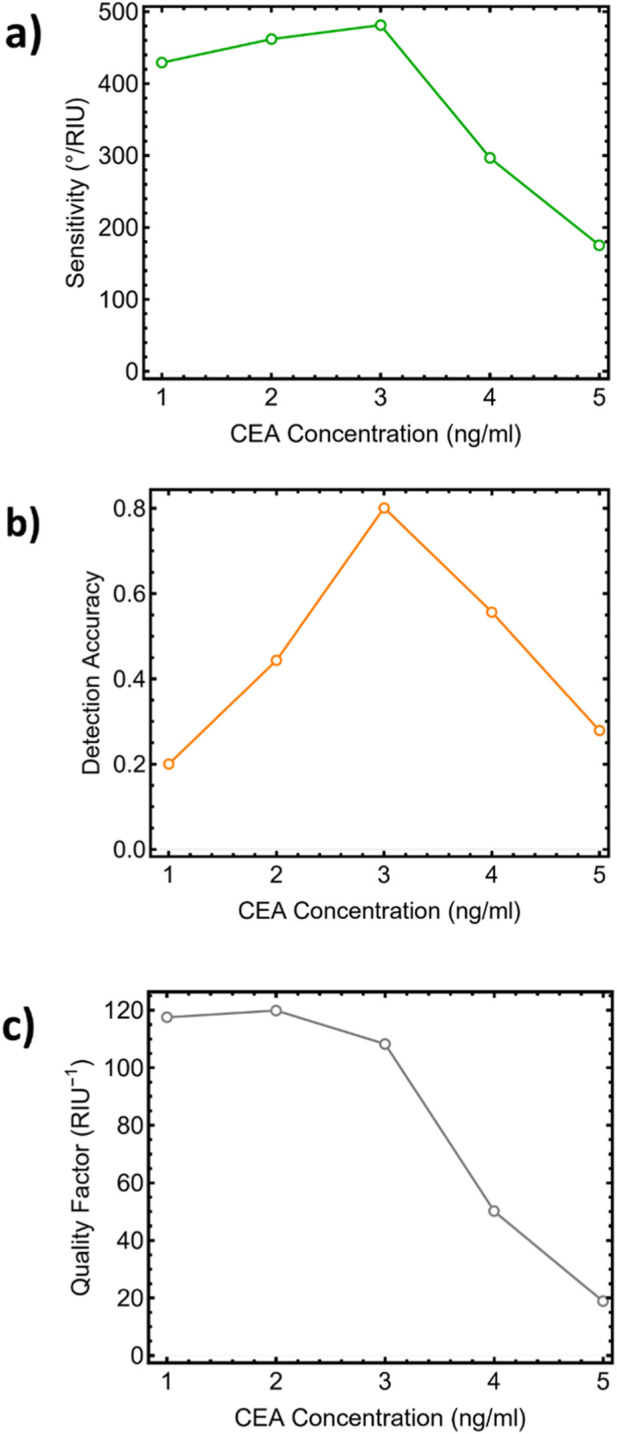
Performance of the CaF_2_/Cu/TiO_2_/G biosensor versus carcinoma concentration: **(a)** Sensitivity (°·RIU^−1^), **(b)** Detection Accuracy (DA), and **(c)** Quality Factor (QF). The optimum response occurs near 3 ng·mL^−1^.

The sensitivity (°/RIU), shown in Figure 11a and Supplementary Table S18, increases with carcinoma concentrations up to 3 ng/mL, reaching a maximum of 481.29°/RIU for Sys_3_ (3 ng/mL). Beyond this point, the sensitivity decreases significantly, suggesting saturation-like behavior due to the nonlinear response of the surface plasmon resonance to high refractive index perturbations. A similar trend is observed for the DA, which reaches its maximum value of 0.80 for Sys_3_ (Figure 11b). This implies optimal optical confinement and a shift in the resonance angle at moderate analyte concentrations. Sys_1_ and Sys_2_ (1–2 ng/mL) show moderate performance with a sensitivity of around 429–461 RIU and a DA in the range of 0.19–0.64, indicating their ability to detect low biomarker loads, but with lower resolution. Sys_5_ (5 ng/mL) exhibits the lowest performance in both metrics due to excessive damping and angular broadening, which reduces the distinction between reflectance dips. In the case of QF, which balances angular sharpness and minimum reflectance, it is also maximized for Sys_2_ (2 ng/mL) and Sys_3_ (3 ng/mL), as shown in Figure 11c and Supplementary Table S18. These systems offer QF values of 119.89 and 103.27 RIU^−1^, respectively. Notably, Sys_4_ and Sys_5_ show drastic drops in QF, reaching only 50.17 and 18.81 RIU^−1^, which correlates with a wider FWHM and less reflectance attenuation, making the signal less distinguishable and therefore more difficult to quantify.

**FIGURE 12:**
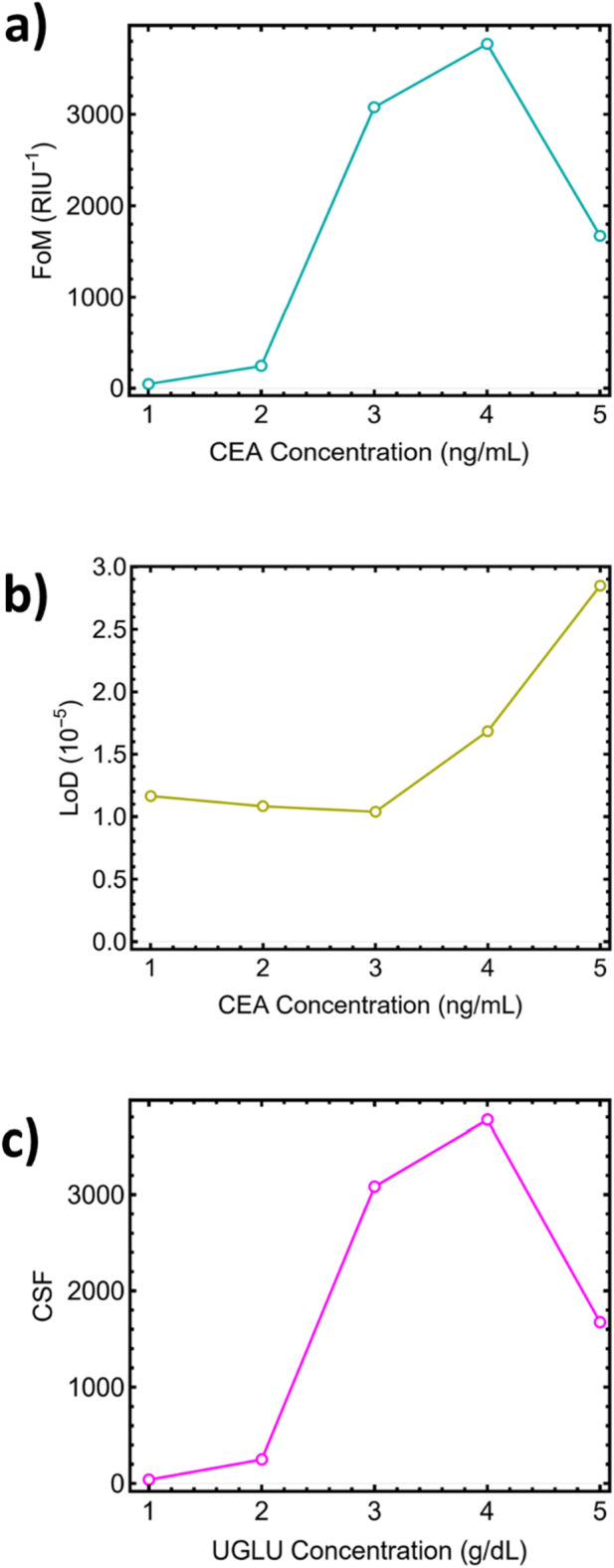
Extended metrics of SPR biosensor systems: **(a)** FoM, **(b)** LoD), and **(c)** Contrast of Signal Factor (CSF), highlighting the superiority of Sys_3_ (3 ng/mL) across all indicators.

The FoM results indicate that it incorporates both sensitivity and angular linewidth. Figure 12a and Supplementary Table S19 show that Sys_3_ and Sys_4_ achieve the highest FoM values, at 3,769.71 and 3,076.25 RIU^−1^, respectively. These results underscore the importance of balancing IR-induced shifts and angular acuity to achieve robust detection performance. Accordingly, the graphene-overlayer case provides a net gain only when the resonance remains sharp. In our implementation a single-layer graphene preserves a narrow FWHM, which sustains high DA and FoM while raising S_RI_; thicker or lossier overlayers would reverse this benefit. Again, Sys_5_ exhibits excessive broadening, reflected in a reduced FoM of 1,673.22 RIU^−1^. In Figure 12b and Supplementary Table S19, Sys_3_ achieves the lowest LoD, at 1.03 × 10^−5^, confirming its excellent resolution in detecting trace carcinoma biomarkers. Conversely, Sys_5_ registers the highest LoD, at 2.84 × 10^−5^, indicating poor detection performance due to signal saturation and broadening. The CSF results on Figure 12c highlight the sharpness and amplitude of the resonance drop-off relative to the background. Sys_3_ again exhibits the highest CSF, at 3,822.99, confirming its excellent signal distinction at optimal carcinoma concentrations. In contrast, Sys_5_ drops to 1,674.34, further corroborating its reduced contrast and poor usability in real-world biodetection scenarios. Consistent with this trade-off, we retain a monolayer graphene overlayer as the preferred configuration to maximize FoM without compromising DA.

From the comparative analysis of all sensor metrics, Sys_3_, corresponding to a carcinoma concentration of 3 ng·mL^−1^, proves to be the most effective configuration. It offers a balanced trade-off between sensitivity (481.29°/RIU), DA (0.80), FoM (3,769.71), and LoD (1.03 × 10^−5^), while maintaining a high CSF (3,822.99). These figures clearly demonstrate that this system achieves an optimal resonance shift with a narrow reflectance dip, which is vital for reliable SPR biodetection. Importantly, while Sys_2_ exhibits slightly lower values in some metrics, it delivers competitive performance with even lower angular broadening and a higher QF. Therefore, for ultra-precision applications where resolution outweighs dynamic range, Sys_2_ is also a strong option. In the case of Sys_5_ (high analyte concentration), performance degradation is observed across all parameters: the reflectance curve flattens, sensitivity decreases, LoD increases, and CSF decreases, likely due to field saturation at the detection interface.

Although a full tolerance study was not undertaken, trends between neighboring tested points indicate that linewidth is more sensitive to t_Cu_ than to t_TiO2_, and that extra graphene layers broaden the resonance; we therefore retain a monolayer to safeguard DA and FoM.

### 3.7 Spatial distribution of the electric field and sensing interface performance

The performance metrics and angular analyses described above are evaluated by the electromagnetic field distribution along the sensor’s multilayer structure. Supplementary Figure S6 presents the spatial profile of the normalized electric-field intensity (∣E∣^2^) as a function of distance from the prism, extending through each layer of the optimized CaF_2_/Cu/TiO_2_/G configuration. This simulation clarifies how plasmonic waves are confined and propagated, and how they interact with carcinoma analytes at varying concentrations. The color-coded vertical bands indicate the Cu, TiO_2_, graphene, and analyte regions. The ∣E∣^2^ profiles are shown for deionized water (reference) and carcinoma concentrations from 1 to 5 ng·mL^−1^. Each curve reveals how the plasmonic field extends from the metal interface into the surrounding dielectric, a key factor governing penetration depth and sensitivity to biomolecular changes.

As shown in Supplementary Figure S6, the ∣E∣^2^ profile exhibits a hotspot at the Graphene/analyte interface and an evanescent tail that extends into the analyte. As carcinoma concentration (and thus refractive index) increases, a larger fraction of the modal energy samples the sensing region, which directly steepens the θ–n response (higher angular refractive-index sensitivity S_RI_; see Section 2.1). In practical terms, stronger near-surface ∣E∣^2^ yields a larger Δθ for a given Δn. The curves at 3–4 ng/mL (Sys_3_, Sys_4_) show the largest decay tail within the analyte, consistent with the superior DA and FoM reported for these configurations, because DA = Δθ/FWHM and FoM = S_RI_(1−R_min_)/FWHM. At 5 ng/mL, the field extends further but the near-interface growth diminishes, indicating the onset of saturation and matching the modest performance gains at this concentration.

The presence of the graphene monolayer and the TiO_2_ spacer plays a fundamental role in shaping this field distribution. Graphene contributes to the enhancement of the local field due to its high surface conductivity and its ability to withstand π–π interactions with biomolecules (Li et al., 2016). Meanwhile, the 8 nm TiO_2_ layer provides a dielectric fit that not only stabilizes the copper layer but also shifts the field maximum away from the lossy metal, improving confinement in the detection region. Combined, these layers provide a custom-designed interface that balances field confinement with analytical accessibility. This customized distribution ensures that even low-concentration biomarkers can modulate the plasmonic resonance, enhancing the sensor’s ability to detect early-stage carcinoma. This field localization also explains the behavior of the DA and the FoM. Because DA = Δθ/FWHM and FoM = S_RI_(1−R_min_)/FWHM (Section 2.1), the simultaneous increase of Δθ driven by the near-surface hotspot, together with impedance matching from the CaF_2_​ prism and the TiO_2_​ spacer that keeps the resonance FWHM narrow, results in higher DA and FoM without excessive linewidth penalties.

When comparing curves, differences in the field profile become appreciable from 2 ng/mL onward. At 3 ng/mL—the operating point that maximizes interface sampling—the decay tail within the analyte is largest among the non-saturating cases, consistent with the observed increases in S_RI_ and in the composite metrics (DA and FoM). At 5 ng/mL, the field extends further but the growth in ∣E∣^2^ near the interface diminishes, indicating the onset of saturation and matching the modest performance gains reported at that concentration.

### 3.8 Comparative analysis with reported SPR biosensors

A comparative analysis was performed with several state-of-the-art sensors published in the literature. The results, summarized in Supplementary Table S19, highlight the significant performance improvements achieved in this work, especially in terms of sensitivity, QF, and DA, crucial parameters for the biodetection of carcinomas. Juwel et al. (2026) presented an SPR configuration that achieves a sensitivity of 393.83°/RIU at a concentration of 5 ng/mL, while (Kumar et al., 2025) achieved 348.07°/RIU with the same analyte concentration.

Our work achieves a maximum sensitivity of 481.29°/RIU at 3 ng/mL, significantly exceeding the maximum values ​​reported in the aforementioned studies. Furthermore, the sensor reaches 429.17°/RIU with only 1 ng/mL, demonstrating its impressive responsiveness to trace levels of carcinoma, a vital characteristic for early diagnosis where antigen levels are low. Khodiae and Heidarzadeh (2025) reported a relatively modest sensitivity of 163.63°/RIU; our platform surpasses this value almost threefold at comparable or lower concentrations. In terms of QF, our sensor achieves a maximum value of 119.89 RU^−1^ at 2 ng/mL, again exceeding the 90.11 RU^−1^ obtained by Juwel et al. and the 53.9 RU^−1^ reported (Rafighirami et al., 2025) using a structure focused on the application of Au.

DA in this study reaches a maximum of 0.80 for 3 ng/mL of carcinoma, which is significantly higher than the 0.22 observed in the design of (Juwel et al., 2026) and the 0.10 in the system of (Khodiae and Heidarzadeh, 2025). This high DA underscores the effectiveness of our design in accurately locating the SPR angle corresponding to the presence of the analyte, a crucial attribute for clinical biodetection applications.

One of the distinctive aspects of our sensor lies in the optimized integration of G and TiO_2_ on Cu, supported on a CaF_2_ substrate. This configuration not only ensures better plasmonic coupling and surface interaction with biomolecules, but also mitigates common drawbacks such as the large resonance drops or low attenuation observed in other architectures. The use of graphene as the end layer introduces a large surface area and excellent biocompatibility, which improves analyte interaction and functionalization potential, especially important for the biodetection of carcinomas. The TiO_2_ acts as a spacer that stabilizes the Cu layer and promotes field enhancement without significantly increasing attenuation losses.

## 4 Limitations

This study presents a computational optimization of a CaF_2_/Cu/TiO_2_/Graphene SPR architecture using the transfer-matrix method under the modeling conditions specified in Section 2; no experimental fabrication or surface/structural characterization was performed. To make the scope explicit and to define a clear path toward prototyping, we outline an integrated verification plan. X-ray photoelectron spectroscopy (XPS) will be employed to confirm graphene’s sp^2^ carbon in the C1s envelope (∼284.5 eV), to assess the copper chemical state via Cu 2p (consistent with metallic Cu and without CuO shake-up satellites at ∼941–944 eV), and to verify the Ti 2p doublet characteristic of TiO_2_. In parallel, X-ray diffraction (XRD) will be used to determine the TiO_2_ phase (e.g., anatase versus rutile) and the preferred crystallographic texture of Cu, while screening for parasitic copper oxides. These structural and chemical verifications will be paired with angle-resolved optical measurements to compare the measured resonance position and linewidth against model predictions. Together, this framework operationalizes the transition from simulation to prototype and specifies the experimental criteria—layer identity, oxidation state, and phase purity—needed to benchmark the modeled performance before device fabrication.

In addition, all metrics reported here (S, DA, FoM, FWHM, LoD) are local to 633 nm; extension to other bands or to wavelength interrogation would require dispersive n(λ),k(λ) and re-optimization of layer thicknesses, as resonance linewidth and contrast can shift with wavelength. Experimental realization may also introduce graphene-transfer defects/residues and added optical loss during surface functionalization, as well as long-term drift from copper oxidation at defects and biofouling; these factors can broaden the resonance and reduce DA/FoM unless mitigated by monolayer-quality transfer, gentle π–π linkers with antifouling steps, edge sealing, and standardized stability/regeneration tests. These limitations do not alter the qualitative trends reported but delineate the operating window and the process controls required for future validation.

## 5 Conclusion

In this work, through systematic layer-by-layer optimization, the potential of this platform for high-performance biodetection was demonstrated, highlighting the synergy between material selection, structural tuning, and optical field enhancement. Key structural components were initially optimized individually. The choice of CaF_2_ as the prism material proved optimal due to its high refractive index and superior light coupling efficiency in the SPR regime. In the study of the metal layer, copper (Cu) was selected instead of noble metals such as gold or silver due to its favorable plasmonic properties and its tunable resonance response in conjunction with the dielectric interlayers. The TiO_2_ dielectric spacer interlayer played a fundamental role in modulating resonance sharpness, improving sensitivity by modifying field confinement at the metal-dielectric interface.

The incorporation of two-dimensional nanomaterials, such as G, GO, rGO, and SWCNT, further improved performance by altering the local refractive index environment and interacting favorably with the evanescent field, presenting more balanced and optimized metrics, including a sensitivity of up to 481.29°/RIU, a DA accuracy of 0.80 degrees^-1^, and a QF of 108.27. A detailed analysis of performance under different carcinoma concentrations (1–5 ng/mL) showed a strong agreement between the modeled refractive index changes and the angular resonance variations. The peak shift of SPR, sensitivity (%), attenuation, and FWHM values at all concentrations indicated a consistent and measurable optical response. The sensor was able to detect Δθ changes of up to 18.10° and exhibited a LoD of only 0.88 × 10^−5^ RIU, demonstrating its suitability for the early identification of biomarkers. This work lays the groundwork for future experimental validation and integration into compact point-of-care SPR diagnostic devices. Follow-up work will implement XPS/XRD verification of layer chemistry and phase and will benchmark the modeled optical response against fabricated devices.

## Data Availability

The original contributions presented in the study are included in the article/Supplementary Material, further inquiries can be directed to the corresponding authors.
