# Supplementary material for: SPR biosensor with a graphene overlayer for carcinoma detection
Source: Front Bioeng Biotechnol. 2026 Feb 23;14:1789453. doi: 10.3389/fbioe.2026.1789453 (PMC12968620; doi:10.3389/fbioe.2026.1789453)
Supplement: Supplementary file 1 [file Supplementaryfile1.docx]

**SUPPLEMENTARY INFORMATION: SPR Biosensor with a Graphene Overlayer for Carcinoma Detection**

Talia Tene^1,^*, Katherine Tixi Gallegos^2^, María José Mendoza Salazar^3^, Lala Gahramanli^4,5^, Rana Khankishiyeva^6,7^, Cristian Vacacela Gomez^8,9,^*

^1^Department of Chemistry, Universidad Técnica Particular de Loja, Loja 110160, Ecuador

^2^Carrera de Ingeniería Química, Facultad de Ciencias, Escuela Superior Politécnica de Chimborazo (ESPOCH), Panamericana Sur km. 1 1/2, Riobamba 060155, Ecuador

^3^Facultad de Ciencias, Grupo de Investigación CIDED, Escuela Superior Politécnica de Chimborazo (ESPOCH), Panamericana Sur km. 1 1/2, Riobamba, 060155, Ecuador.

^4^Nano Research Laboratory, Excellent Center, Baku State University, Academic Zahid Khalilov St. 23, Baku AZ1148, Azerbaijan

^5^Faculty of Physics, Chemical Physics of Nanomaterials, Baku State University, Baku, Azerbaijan

^6^Institute of Radiation Problems, Ministry of Science and Education of the Republic of Azerbaijan, Baku, AZ1143, Azerbaijan

^7^Department of Physics and Chemistry, Azerbaijan University of Architecture and Construction, Baku, AZ1073, Azerbaijan

^8^Department of Physics, University of Calabria, Via P. Bucci, Cubo 33C, Rende 87036, Italy

^9^Universidad Ecotec, Km. 13.5 Samborondón, Samborondón, EC092302, Ecuador

*Correspondence: [tbtene@utpl.edu.ec](mailto:tbtene@utpl.edu.ec) (T.T.) [cristianisaac.vacacelagomez@fis.unical.it](mailto:cristianisaac.vacacelagomez@fis.unical.it) (C.V.G.)

1. **Numerical Modeling approach**

The reflective intensity of the proposed *N^th^*-layer sensor model is calculated using the TMM [37,38]. Then, the analysis of the sensor considers boundary conditions for the tangential component, with initial limit Z = Z_1_ = 0, and final limit Z_n-1_, giving the following expression:

| $\left[ \begin{matrix} E_{1} \\ H_{1} \end{matrix} \right]=M_{ij}\left[ \begin{matrix} E_{N-1} \\ H_{N-1} \end{matrix} \right]$ | (S1) |
| --- | --- |

In Equation (S1), *E_1_*, *E_N-1_*, *V_1_*, and *V_N-1_* represents the tangential components of the electric and magnetic fields for the initial and *N^th^* layer, respectively. M*_ij_* indicates the transfer matrix characteristics of the *N^th^* layer model. The transfer matrix can be computed as:

| $M_{ij}=\left[ \prod_{k=2}^{N-1} M_{k} \right]_{ij}=\left[ \begin{matrix} M_{11} & M_{12} \\ M_{21} & M_{22} \end{matrix} \right]$ | (S2) |
| --- | --- |

With

| $M_{k}=\left[ \begin{matrix} \cos\beta_{k} & (-i sin \beta_{k})/q_{k} \\ -i q_{k}\sin\beta_{k} & \cos\beta_{k} \end{matrix} \right]$ | (S3) |
| --- | --- |

Denoting

| $\beta_{k}=\frac{2\pi d_{k}}{\lambda_{0}}\sqrt{\varepsilon_{k}-n_{1}^{2}\sin^{2} \theta}$ | (S4) |
| --- | --- |

And

| $q_{k}=\frac{\sqrt{\varepsilon_{k}-n_{1}^{2}\sin^{2} \theta}}{\varepsilon_{k}}$ | (S5) |
| --- | --- |

in Equation (S3)-(S5),$\lambda_{0}$ represents the wavelength of the incident light, $n_{1}$ is the refractive index, $\varepsilon_{k}$ represents the dielectric constant, $\beta_{k}$ represents the phase constant, $\theta$ represents the entrance angle, and $d_{k}$ represents the depth of the $k^{th}$ layer. For comparison with experiments, we adopt the use of He-Ne laser with $\lambda_{0}=633$ nm.

After straightforward computations, the total reflection of the *N^th^*-layer model can be expressed as:

| $R=\left\vert\frac{\left( M_{11}+M_{12} q_{N} \right)q_{1}-\left( M_{21}+M_{22} q_{N} \right)}{\left( M_{11}+M_{12} q_{N} \right)q_{1}+\left( M_{21}+M_{22} q_{N} \right)} \right\vert^{2}$ | (S6) |
| --- | --- |

By using Equation (S6), the reflectance as a function of the angle of incidence (SPR curve) can be calculated.

We now move on the main performance metric of the proposed sensors. The first parameter is the sensitivity enhancement regarding the baseline sensors after/before pathogen adsorption, denoted as:

| $\Delta S_{RI}^{after}=\frac{(S_{RI}^{after}-S_{RI}^{before})}{S_{RI}^{before}}$ | (S7) |
| --- | --- |

1. **Supplementary Figures**

**
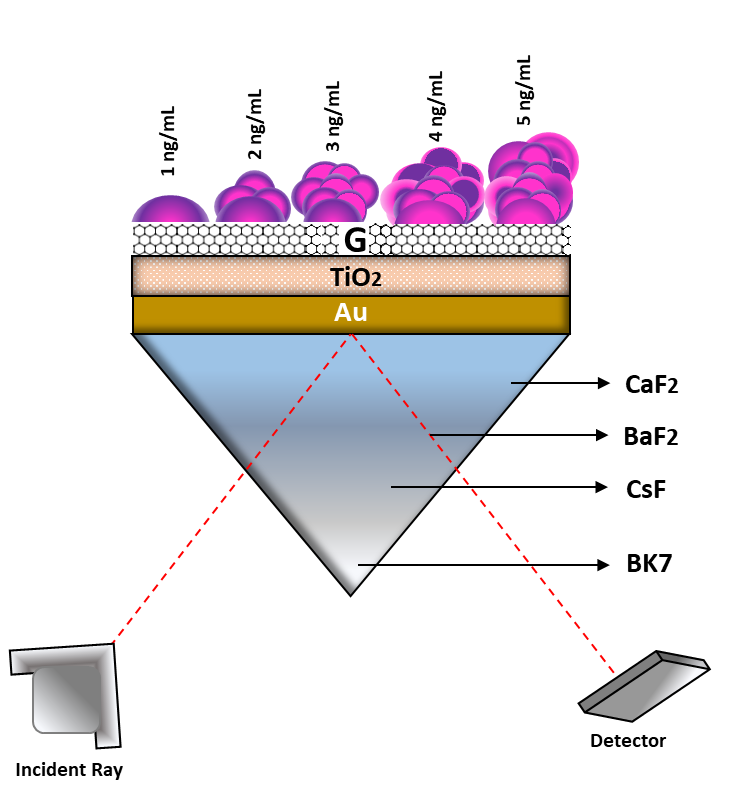
**

**Scheme S1.** Kretschmann SPR sensor architecture for low-concentration carcinoma biomarker detection. The multilayer stack includes a high-index prism (BK7, CsF, BaF₂, or CaF₂), a Cu plasmonic layer, a TiO₂ spacer, and optional 2D carbon-based coatings. Angular interrogation (θ) at λ = 633 nm under TM polarization enables detection via resonance shifts.


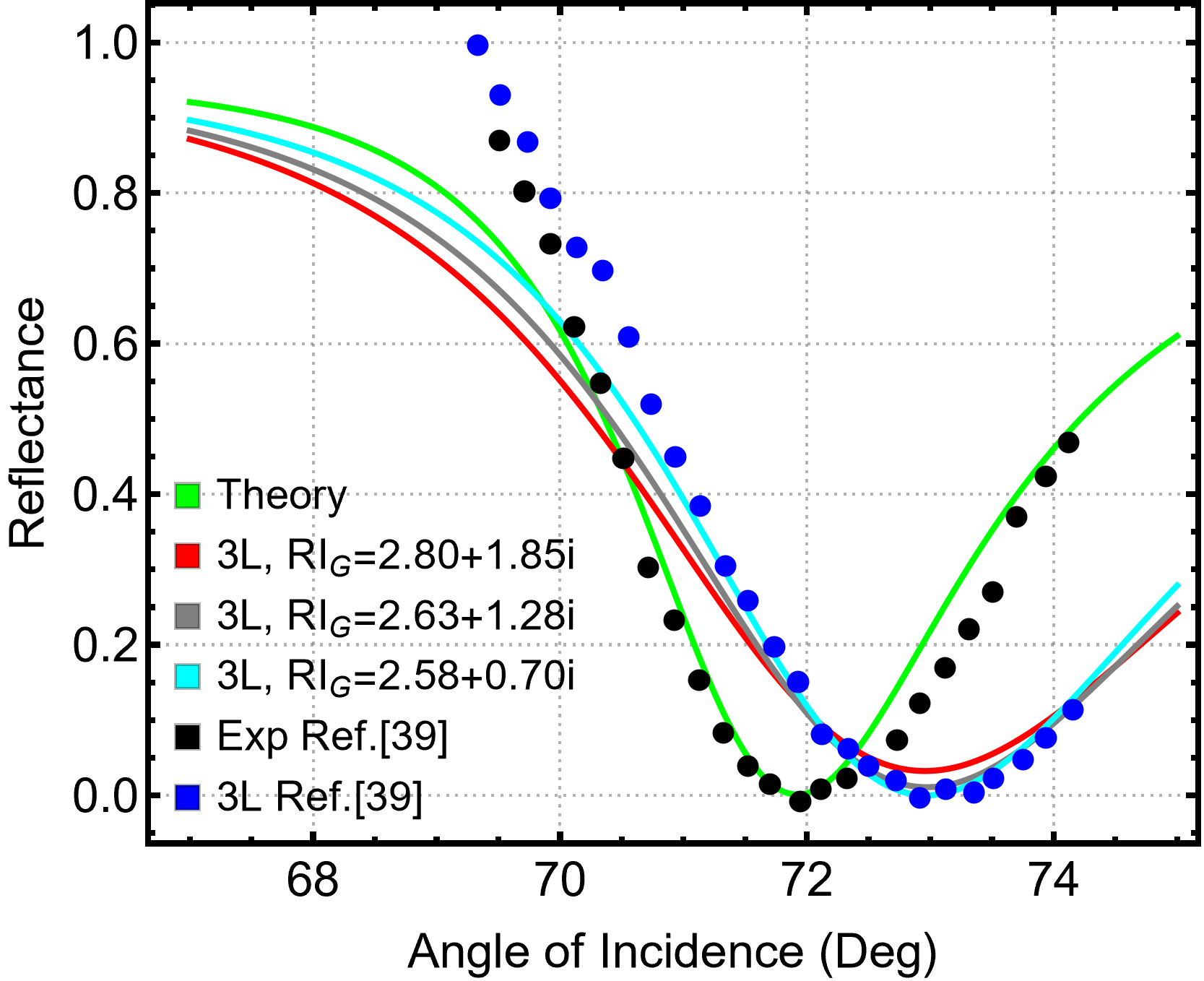


**Figure S1.** TMM validation: angle-dependent reflectance R(θ) of a three-layer model compared with experimental data from Ref. [39], illustrating the typical line shape and agreement with the literature.


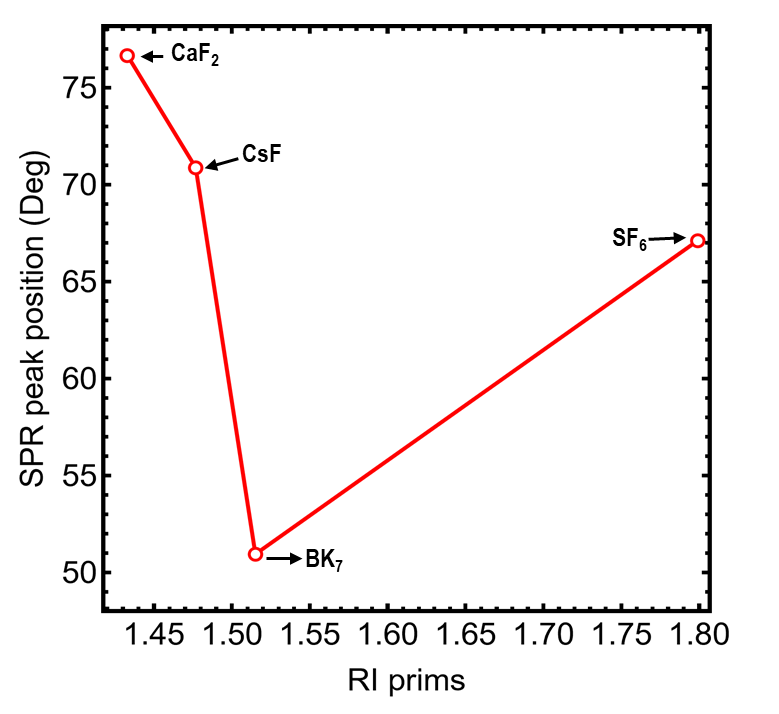


**Figure S2.** Relationship between SPR peak angle and refractive index of the prism.


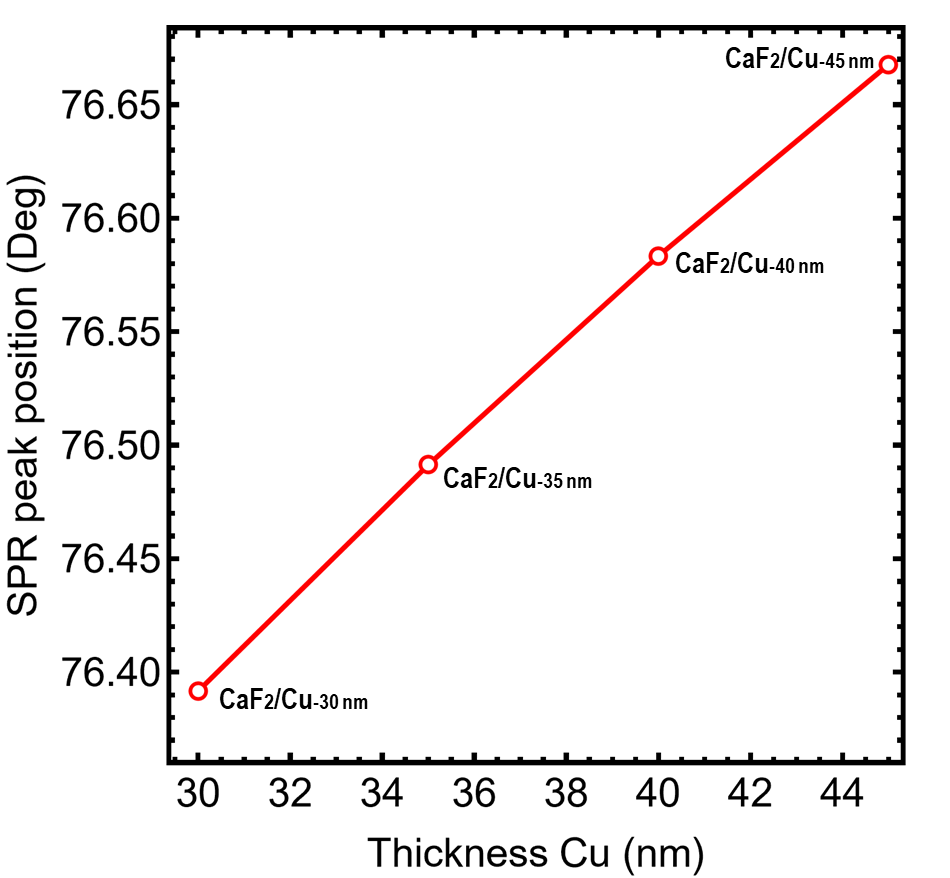


**Figure S3.** SPR resonance angle as a function of Cu thickness


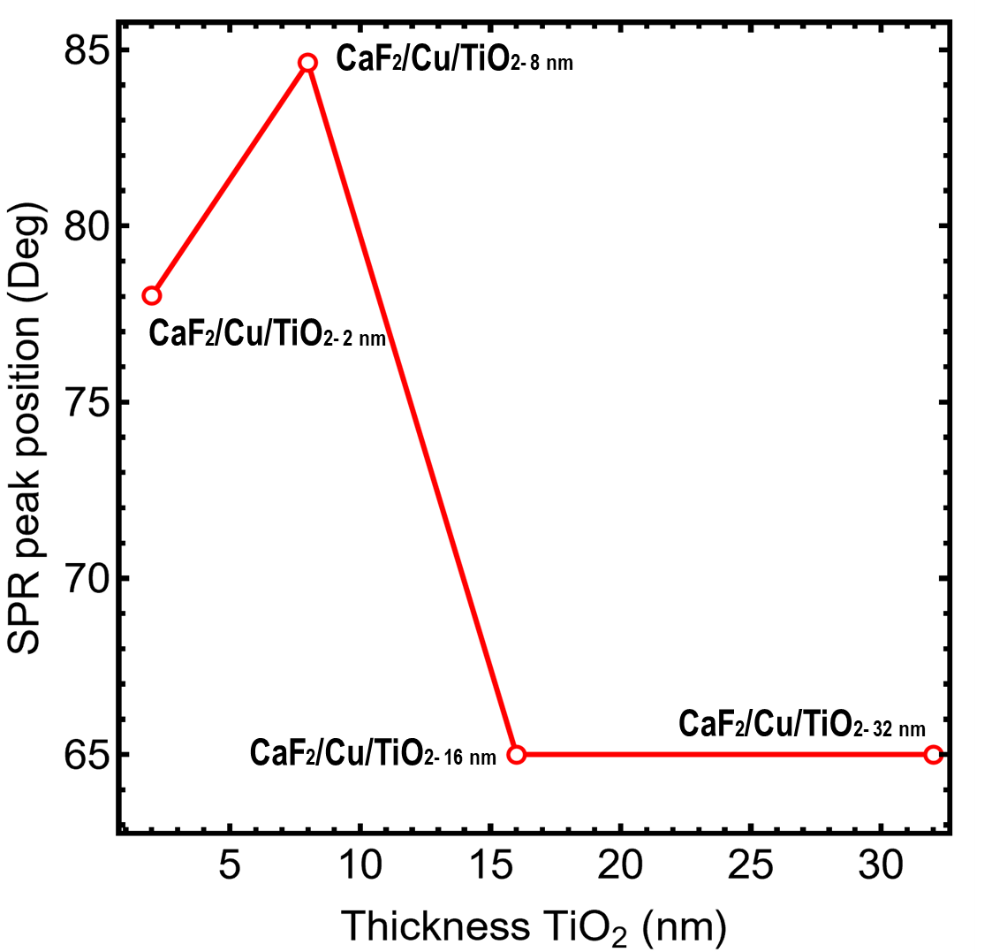


**Figure S4.** SPR peak angle vs. TiO₂ layer thickness.


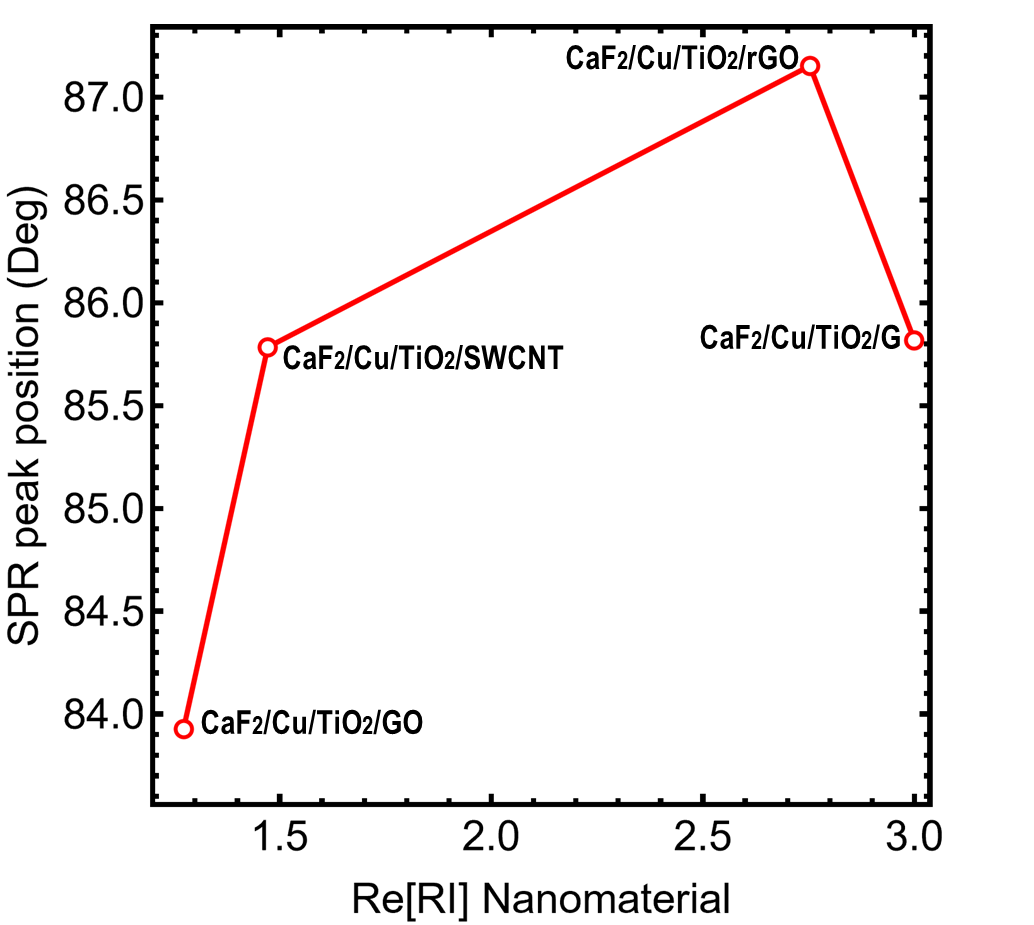


**Figure S5.** Resonance angle as a function of nanomaterial refractive index

**
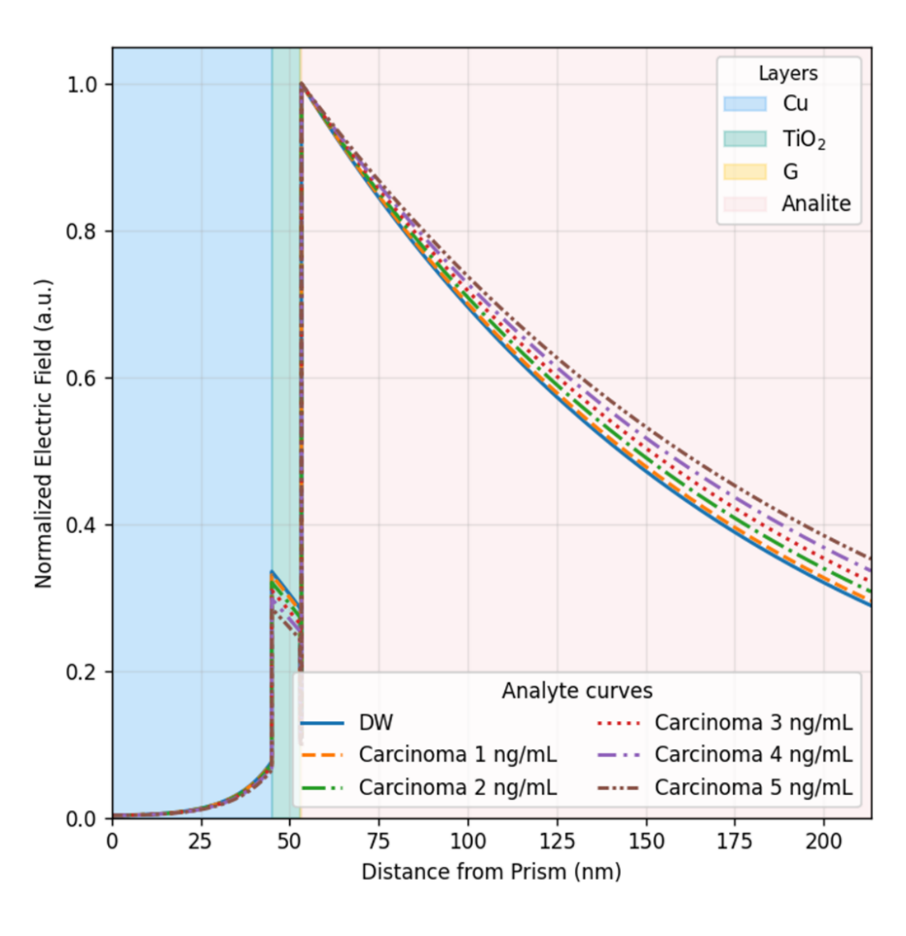
**

**Figure 6S.** Spatial distribution of the normalized electric field across the CaF₂/Cu/TiO₂/G sensor structure for varying carcinoma concentrations (1–5 ng/mL) and DW.

1. **Supplementary Tables**

**Table S1.** Tested SPR systems for performance comparison. Each system varies in substrate and analyte configuration at 1 ng/mL carcinoma concentration.

| **Code** | **Full Name** | **Short Name** |
| --- | --- | --- |
| Sys_0-Bk7_ | BK_7_/Copper/Water | BK_7_/Cu/H_2_O |
| Sys_1-CaF2_ | CaF_2_/Copper/Carcinoma-1 ng/ml | CaF_2_/Cu/Carcinoma-_1 ng/ml_ |
| Sys_2-CsF_ | CsF/Copper/Carcinoma-1 ng/ml | CsF/Cu/Carcinoma-_1 ng/ml_ |
| Sys_3-SF6_ | SF_6_/Copper/Carcinoma-1 ng/ml | SF_6_/Cu/Carcinoma-_1 ng/ml_ |
| Sys_4-BK7_ | BK_7_/Copper/Carcinoma-1 ng/ml | BK_7_/Cu/Carcinoma-_1 ng/ml_ |

**Table S2.** Optical properties and thicknesses of materials used in the SPR sensor simulations. Refractive indices are taken from literature references.

| **Material** | **Refractive Index** | **Thickness (nm)** | **Ref.** |
| --- | --- | --- | --- |
| BK_7_ | 1.5151 | --- | [40] |
| CaF_2_ | 1.4329 | --- | [40] |
| CsF | 1.4768 | --- | [40] |
| SF_6_ | 1.7990 | --- | [40] |
| Copper (Cu) | 0.0369 + 4.5393i | 45.0 | [40] |
| Titanium Dioxide (TiO_2_) | 1.99 | 2.00 | [41] |
| Graphene Oxide (GO) | 1.27280 + 0.00392835 i | 2.55 | [42] |
| Semiconducting Single-Wall Carbon Nanotube (sSWCNT) | 2.0394 + 0.08000000 i | 2.04 | [43] |
| Reduced graphene oxide (rGO) | 2.75291 + 0.410475i | 1.41 | [44] |
| Graphene (G) | 3.0 + 1.14910600 i | 0.34 | [40] |
| Water medium (H_2_O) | 1.3300 | --- | [45] |
| Carcinoma (1 ng/ml) | 1.3337 | --- | [46] |

**Table S3.** Performance metrics of prism-based configurations: resonance position, angular shift, sensitivity, attenuation, and FWHM.

| **Code** | **SPR Peak position** | $\boldsymbol{\Delta\theta}$ **(Deg)** | **Sensitivity Enhancement (%)** | **Attenuation (%)** | **FWHM** |
| --- | --- | --- | --- | --- | --- |
| Sys_1-CaF2_ | 76.66 | 9.94 | 14.90 | 27.53 | 1.56 |
| Sys_2-CsF_ | 70.87 | 4.15 | 6.22 | 28.71 | 1.19 |
| Sys_3-SF6_ | 50.94 | 15.78 | 23.65 | 23.75 | 0.49 |
| Sys_4-BK7_ | 67.11 | 0.39 | 0.58 | 27.93 | 1.00 |

**Table S4.** Refractive index values for prism materials and corresponding SPR peak angles.

| **Code** | **RI prism** | **SPR Peak position** |
| --- | --- | --- |
| Sys_1-CaF2_ | 1.4329 | 76.66 |
| Sys_2-CsF_ | 1.4768 | 70.87 |
| Sys_3-SF6_ | 1.7990 | 50.94 |
| Sys_4-BK7_ | 1.5151 | 67.11 |

**Table S5.** Description of simulated multilayer systems including Cu thickness and analyte conditions

| **Code** | **Full Name** | **Short Name** |
| --- | --- | --- |
| Sys_0-Cu_ | CaF_2_/Copper/Water | CaF_2_/Cu/H_2_O |
| Sys_1-Cu-45nm_ | CaF_2_/Copper _45nm_/Carcinoma-_1ng/ml_ | CaF_2_/Cu_-45nm_/Carcinoma_-1ng/ml_ |
| Sys_2-Cu-40nm_ | CaF_2_/Copper _40nm_/Carcinoma-_1ng/ml_ | CaF_2_/Cu_-40nm_/Carcinoma_-1ng/ml_ |
| Sys_3-Cu-35nm_ | CaF_2_/Copper _35nm_/Carcinoma-_1ng/ml_ | CaF_2_/Cu_-35nm_/Carcinoma_-1ng/ml_ |
| Sys_4-Cu-30nm_ | CaF_2_/Copper _30nm_/Carcinoma-_1ng/ml_ | CaF_2_/Cu_-30nm_/Carcinoma_-1ng/ml_ |

**Table S6.** Optical performance metrics: resonance angle, Δθ, sensitivity, attenuation, and FWHM.

| **Code** | **SPR Peak position** | $\boldsymbol{\Delta\theta}$ **(Deg)** | **Sensitivity Enhancement (%)** | **Attenuation (%)** | **FWHM** |
| --- | --- | --- | --- | --- | --- |
| Sys_1-Cu-45nm_ | 76.66 | 0.69 | 0.90 | 27.53 | 1.52 |
| Sys_2-Cu-40nm_ | 76.58 | 0.60 | 0.79 | 46.30 | 2.19 |
| Sys_3-Cu-35nm_ | 76.49 | 0.51 | 0.67 | 63.00 | 3.25 |
| Sys_4-Cu-30nm_ | 76.39 | 0.41 | 0.54 | 75.91 | 4.95 |

**Table S7.** Refractive index (real part) of copper and corresponding SPR peak position for each configuration.

| **Code** | **RI metal (real part)** | **SPR Peak position** |
| --- | --- | --- |
| Sys_1-Cu-45nm_ | 0.0369 | 76.66 |
| Sys_2-Cu-40nm_ |  | 76.58 |
| Sys_3-Cu-35nm_ |  | 76.49 |
| Sys_4-Cu-30nm_ |  | 76.39 |

**Table S8.** Descriptions of simulated multilayer systems with varying TiO₂ thickness and analyte conditions.

| **Code** | **Full Name** | **Short Name** |
| --- | --- | --- |
| Sys_0-Cu+TiO2_ | CaF_2_/Copper/Titanium Dioxide/Water | CaF_2_/Cu/TiO_2_/H_2_O |
| Sys_1-Cu+TiO2-2nm_ | CaF_2_/Copper/Titanium Dioxide_-2nm_/Carcinoma_-1ng/ml_ | CaF_2_/Cu/TiO_2-2nm_/Carcinoma_-1ng/ml_ |
| Sys_2-Cu+TiO2-8nm_ | CaF_2_/Copper/Titanium Dioxide_-8nm_/Carcinoma_-1ng/ml_ | CaF_2_/Cu/TiO_2-8nm_/Carcinoma_-1ng/ml_ |
| Sys_3-Cu+TiO2-16nm_ | CaF_2_/Copper/Titanium Dioxide_-16nm_/Carcinoma_-1ng/ml_ | CaF_2_/Cu/TiO_2-16nm_/Carcinoma_-1ng/ml_ |
| Sys_4-Cu+TiO2-32nm_ | CaF_2_/Copper/Titanium Dioxide_-32nm_/Carcinoma_-1ng/ml_ | CaF_2_/Cu/TiO_2-32nm_/Carcinoma_-1ng/ml_ |

**Table S9.** Key optical metrics: resonance position, Δθ, sensitivity, attenuation, and FWHM.

| **Code** | **SPR Peak position** | $\boldsymbol{\Delta\theta}$ **(Deg)** | **Sensitivity Enhancement (%)** | **Attenuation (%)** | **FWHM** |
| --- | --- | --- | --- | --- | --- |
| Sys_1-Cu+TiO2-2nm_ | 78.01 | 0.75 | 0.98 | 26.21 | 1.74 |
| Sys_2-Cu+TiO2-8nm_ | 84.62 | 7.36 | 9.53 | 9.07 | 2.71 |
| Sys_3-Cu+TiO2-16nm_ | 65.00 | 12.25 | 15.86 | 94.72 | 3.42x10^14^ |
| Sys_4-Cu+TiO2-32nm_ | 65.00 | 12.25 | 15.86 | 95.53 | 0.56 |

**Table S10.** SPR peak angles and corresponding real part of the Cu refractive index.

| **Code** | **RI metal (real part)** | **SPR Peak position** |
| --- | --- | --- |
| Sys_1-Cu+TiO2-2nm_ | 1.99 | 78.01 |
| Sys_2-Cu+TiO2-8nm_ |  | 84.62 |
| Sys_3-Cu+TiO2-16nm_ |  | 65.00 |
| Sys_4-Cu+TiO2-32nm_ |  | 65.00 |

**Table S11.** Descriptions of each system with corresponding nanomaterial and analyte conditions.

| **Code** | **Full Name** | **Short Name** |
| --- | --- | --- |
| Sys_0-CaF2/Cu/TiO2/G/H2O_ | CaF_2_/Copper/Titanium Dioxide/Graphene/Water | CaF_2_/Cu/TiO_2_/G/H_2_O |
| Sys_1-CaF2/Cu/TiO2/GO/Carcinoma-1ng/ml_ | CaF_2_/Copper/Titanium Dioxide/Graphene Oxide/Carcinoma_-1ng/ml_ | CaF_2_/Cu/TiO_2_/GO/Carcinoma_-1ng/ml_ |
| Sys_2-CaF2/Cu/TiO2/sSWCNT/Carcinoma-1ng/ml_ | CaF_2_/Copper/Titanium Dioxide/semiconducting-single-wall carbon nanotubes/Carcinoma_-1ng/ml_ | CaF_2_/Cu/TiO_2_/sSWCNT/Carcinoma_-1ng/ml_ |
| Sys_3-CaF2/Cu/TiO2/rGO/Carcinoma-1ng/ml_ | CaF_2_/Copper/Titanium Dioxide/reduced graphene oxide/Carcinoma_-1ng/ml_ | CaF_2_/Cu/TiO_2_/rGO/Carcinoma_-1ng/ml_ |
| Sys_4-CaF2/Cu/TiO2/G/Carcinoma-1ng/ml_ | CaF_2_/Copper/Titanium Dioxide/graphene/Carcinoma_-1ng/ml_ | CaF_2_/Cu/TiO_2_/G/Carcinoma_-1ng/ml_ |

**Table S12.** SPR metrics: peak position, Δθ, sensitivity, attenuation, and FWHM for each nanomaterial.

| **Code** | **SPR Peak position** | $\boldsymbol{\Delta\theta}$ **(Deg)** | **Sensitivity Enhancement (%)** | **Attenuation (%)** | **FWHM** |
| --- | --- | --- | --- | --- | --- |
| Sys_1-CaF2/Cu/TiO2/GO/Carcinoma-1ng/ml_ | 83.92 | 0.17 | 0.20 | 9.14 | 2.69 |
| Sys_2-CaF2/Cu/TiO2/sSWCNT/Carcinoma-1ng/ml_ | 85.78 | 1.68 | 1.99 | 3.22 | 3.89 |
| Sys_3-CaF2/Cu/TiO2/rGO/Carcinoma-1ng/ml_ | 87.15 | 3.04 | 3.62 | 71.66 | 6.21 |
| Sys_4-CaF2/Cu/TiO2/G/Carcinoma-1ng/ml_ | 85.81 | 1.71 | 2.03 | 3.01 | 3.85 |

**Table S13.** Refractive index properties of nanomaterials and resulting SPR peak angles

| **Code** | **RI nanomaterial** | **Im** | **SPR Peak position** |
| --- | --- | --- | --- |
| Sys_1-CaF2/Cu/TiO2/GO/Carcinoma-1ng/ml_ | 1.27280 | 0.00392835 | 83.92 |
| Sys_2-CaF2/Cu/TiO2/sSWCNT/Carcinoma-1ng/ml_ | 2.0394 | 0.08 | 85.78 |
| Sys_3-CaF2/Cu/TiO2/rGO/Carcinoma-1ng/ml_ | 2.75291 | 0.410475 | 87.15 |
| Sys_4-CaF2/Cu/TiO2/G/Carcinoma-1ng/ml_ | 3.0 | 1.149106 | 85.81 |

**Table S14.** Refractive index (RI) values corresponding to different carcinoma concentrations (1–5 ng/mL) used to simulate biological variation in the sensing environment.

| **Carcinoma (ng/ml)** | **RI** |
| --- | --- |
| 1 | 1.3337 |
| 2 | 1.3374 |
| 3 | 1.3411 |
| 4 | 1.3448 |
| 5 | 1.3485 |

**Table S15.** Optical constants and thicknesses of materials used in the CaF₂/Cu/TiO₂/G sensor configuration, including the prism, metal, dielectric spacer, nanomaterial overlayer, deionized water, and carcinoma solutions.

| **Material** | **Refractive Index** | **Thickness (nm)** |
| --- | --- | --- |
| CaF_2_ | 1.4329 | --- |
| Copper (Cu) | 0.0369 + 4.5393i | 45.0 |
| Titanium Dioxide (TiO_2_) | 1.99 | 8.00 |
| Graphene (G) | 3.0 + 1.1491i | 0.34 |
| Deionized water (DIW) | 1.331700 | --- |
| Carcinoma -1ng/ml | 1.333700 | --- |
| Carcinoma -2ng/ml | 1.337400 | --- |
| Carcinoma -3ng/ml | 1.341100 | --- |
| Carcinoma -4ng/ml | 1.344800 | --- |
| Carcinoma -5ng/ml | 1.348500 | --- |

**Table S16.** Performance metrics of the optimized SPR biosensor (CaF₂/Cu/TiO₂/G) at different carcinoma concentrations: SPR peak position, angular shift (Δθ), sensitivity enhancement, attenuation, and FWHM.

| **Code** | **SPR Peak position** | $\boldsymbol{\Delta\theta}$ **(Deg)** | **Sensitivity Enhancement (%)** | **Attenuation (%)** | **FWHM** |
| --- | --- | --- | --- | --- | --- |
| Sys_1-CaF2/Cu/TiO2/G/DIW_ | 84.83 | 0.72 | 0.86 | 0.62 | 3.65 |
| Sys_2-CaF2/Cu/TiO2/G/1 ng/ml_ | 85.81 | 1.70 | 2.03 | 3.01 | 3.85 |
| Sys_3-CaF2/Cu/TiO2/G/2 ng/ml_ | 87.66 | 3.56 | 4.23 | 29.41 | 4.44 |
| Sys_4-CaF2/Cu/TiO2/G/3 ng/ml_ | 87.39 | 3.29 | 3.91 | 76.13 | 5.91 |
| Sys_5-CaF2/Cu/TiO2/G/4 ng/ml_ | 86.70 | 2.59 | 3.08 | 89.94 | 9.32 |
| Sys_6-CaF2/Cu/TiO2/G/5 ng/ml_ | 66.00 | 18.10 | 21.52 | 93.97 | 28.11 |

**Table S17.** Correlation between refractive index changes induced by carcinoma concentration and the corresponding SPR peak positions of the sensor, highlighting the system’s refractive index sensitivity.

| **Code** | **RI UGLU** | **SPR Peak position** |
| --- | --- | --- |
| Sys_1-CaF2/Cu/TiO2/G/DIW_ | 1.331700 | 84.83 |
| Sys_2-CaF2/Cu/TiO2/G/1 ng/ml_ | 1.333700 | 85.81 |
| Sys_3-CaF2/Cu/TiO2/G/2 ng/ml_ | 1.337400 | 87.66 |
| Sys_4-CaF2/Cu/TiO2/G/3 ng/ml_ | 1.341100 | 87.39 |
| Sys_5-CaF2/Cu/TiO2/G/4 ng/ml_ | 1.344800 | 86.70 |
| Sys_6-CaF2/Cu/TiO2/G/5 ng/ml_ | 1.348500 | 66.00 |

**Table S18.** Sensitivity, detection accuracy, and quality factor values for each biosensor system as a function of modeled carcinoma concentrations.

| **Modeled UGLU concentrations** | **Modeled RI** | ***S* (**$\boldsymbol{^{\circ}/RIU}$**)** | **DA** | **QF (*RIU*^-1^)** |
| --- | --- | --- | --- | --- |
| Carcinoma 1 ng/ml | 1.333700 | 429.17 | 0.19 | 117.51 |
| Carcinoma 2 ng/ml | 1.337400 | 461.83 | 0.44 | 119.89 |
| Carcinoma 3 ng/ml | 1.341100 | 481.29 | 0.80 | 108.27 |
| Carcinoma 4 ng/ml | 1.344800 | 296.64 | 0.55 | 50.17 |
| Carcinoma 5 ng/ml | 1.348500 | 175.45 | 0.27 | 18.81 |

**Table S19.** Quantitative values of figure of merit, limit of detection, and contrast signal factor derived from modeled RI shifts for carcinoma concentrations between 1–5 ng/ml

| **Modeled UGLU concentrations** | **Modeled RI** | **FoM (*RIU*^-1^)** | **LoD (10^-5^)** | **CSF** |
| --- | --- | --- | --- | --- |
| Carcinoma 1 ng/ml | 1.333700 | 44.54 | 1.16 | 38.18 |
| Carcinoma 2 ng/ml | 1.337400 | 241.32 | 1.08 | 247.93 |
| Carcinoma 3 ng/ml | 1.341100 | 3076.25 | 1.03 | 3082.39 |
| Carcinoma 4 ng/ml | 1.344800 | 3769.71 | 1.68 | 3772.62 |
| Carcinoma 5 ng/ml | 1.348500 | 1673.22 | 2.84 | 1674.34 |

**Table S19.** Comparison of the proposed SPR biosensor with reported designs in terms of structure, analyte concentration, sensitivity (°/RIU), quality factor (QF), and detection accuracy (DA).

| **Ref.** | **Structure** | **Conc. (ng ml⁻¹)** | **Sensitivity (°/RIU)** | **QF** | **DA** |
| --- | --- | --- | --- | --- | --- |
| Juwel et al. [48] | CaF_2_/MgO/Ag/ZnSe/Graphene | 5 | 393.83 | 90.11 | 0.22 |
| Kumar et al. [49] | CaF_2_/Cu/BP/Franckeite | 5 | 348.07 | -- | -- |
| Khodiae et al. [50] | BK7/Ag/Ni/ZnSe | -- | 163.63 | -- | 0.10 |
| Rafighirami et al. [46] | Au/SiO₂/Graphene | 3 | 4.306 THz/RIU | 53.9 | -- |
| This work | CaF₂/Cu/TiO_2_/Graphene | 1 | 429.17 | 117.51 | 0.19 |
| This work | CaF₂/Cu/TiO_2_/Graphene | 2 | 461.83 | 119.89 | 0.44 |
| This work | CaF₂/Cu/TiO_2_/Graphene | 3 | 481.29 | 108.27 | 0.80 |
| This work | CaF₂/Cu/TiO_2_/Graphene | 4 | 296.64 | 50.17 | 0.55 |
| This work | CaF₂/Cu/TiO_2_/Graphene | 5 | 175.45 | 18.81 | 0.27 |
